# Exposure to Bisphenol A and Phthalates during Pregnancy and Ultrasound Measures of Fetal Growth in the INMA-Sabadell Cohort

**DOI:** 10.1289/ehp.1409190

**Published:** 2015-07-21

**Authors:** Maribel Casas, Damaskini Valvi, Ana Ballesteros-Gomez, Mireia Gascon, Mariana F. Fernández, Raquel Garcia-Esteban, Carmen Iñiguez, David Martínez, Mario Murcia, Nuria Monfort, Noelia Luque, Soledad Rubio, Rosa Ventura, Jordi Sunyer, Martine Vrijheid

**Affiliations:** 1 Centre for Research in Environmental Epidemiology (CREAL), Barcelona, Spain; 2 Universitat Pompeu Fabra (UPF), Barcelona, Spain; 3 CIBER Epidemiología y Salud Pública (CIBERESP), Madrid, Spain; 4 Department of Analytical Chemistry, University of Cordoba, Cordoba, Spain; 5 ISGlobal, Barcelona Centre for International Health Research (CRESIB), Hospital Clínic–Universitat de Barcelona, Barcelona, Spain; 6 Instituto de Investigación Biosanitaria de Granada (Granada.ibs), University of Granada, Granada, Spain; 7 Foundation for the Promotion of Health and Biomedical Research in the Valencian Region, FISABIO/CSISP, Valencia, Spain; 8 Universidad de Valencia, Valencia, Spain; 9 Hospital del Mar Medical Research Institute (IMIM), Barcelona, Spain

## Abstract

**Background:**

Prenatal exposure to bisphenol A (BPA) and phthalates may affect fetal growth; however, previous findings are inconsistent and based on few studies.

**Objectives:**

We assessed whether prenatal exposure to BPA and phthalates was associated with fetal growth in a Spanish birth cohort of 488 mother–child pairs.

**Methods:**

We measured BPA and eight phthalates [four di(2-ethylhexyl) phthalate metabolites (DEHPm), mono-benzyl phthalate (MBzP), and three low-molecular-weight phthalate metabolites (LMWPm)] in two spot-urine samples collected during the first and third trimester of pregnancy. We estimated growth curves for femur length (FL), head circumference (HC), abdominal circumference (AC), biparietal diameter (BPD), and estimated fetal weight (EFW) during pregnancy (weeks 12–20 and 20–34), and for birth weight, birth length, head circumference at birth, and placental weight.

**Results:**

Overall, results did not support associations of exposure to BPA or DEHPm during pregnancy with fetal growth parameters. Prenatal MBzP exposure was positively associated with FL at 20–34 weeks, resulting in an increase of 3.70% of the average FL (95% CI: 0.75, 6.63%) per doubling of MBzP concentration. MBzP was positively associated with birth weight among boys (48 g; 95% CI: 6, 90) but not in girls (–27 g; 95% CI: –79, 25) (interaction p-value = 0.04). The LMWPm mono-n-butyl phthalate (MnBP) was negatively associated with HC at 12–20 pregnancy weeks [–4.88% of HC average (95% CI: –8.36, –1.36%)].

**Conclusions:**

This study, one of the first to combine repeat exposure biomarker measurements and multiple growth measures during pregnancy, finds little evidence of associations of BPA or phthalate exposures with fetal growth. Phthalate metabolites MBzP and MnBP were associated with some fetal growth parameters, but these findings require replication.

**Citation:**

Casas M, Valvi D, Ballesteros-Gomez A, Gascon M, Fernández MF, Garcia-Esteban R, Iñiguez C, Martínez D, Murcia M, Monfort N, Luque N, Rubio S, Ventura R, Sunyer J, Vrijheid M. 2016. Exposure to bisphenol A and phthalates during pregnancy and ultrasound measures of fetal growth in the INMA-Sabadell cohort. Environ Health Perspect 124:521–528; http://dx.doi.org/10.1289/ehp.1409190

## Introduction

Bisphenol A (BPA) and phthalates are a class of synthetic chemicals produced and used in large quantities worldwide and present in many kind of articles including plastics, cosmetics, carpets, building materials, toys, or cleaning products (Koch and Calafat 2009). In 1999, the European Union banned the use of some phthalates in the manufacture of toys and childcare articles, and in 2011, the use of BPA was banned in infant feeding bottles (European Commission 2005, 2011). In the United States, environmental and public health organizations have conducted numerous campaigns to reduce their use in consumer products and concentrations in the general population of some phthalates have started to decline (Zota et al. 2014). Diet is the predominant source of BPA and high-molecular-weight phthalates (HMWP) (Rudel et al. 2011; Wormuth et al. 2006), whereas personal care products are the major source of the low-molecular-weight phthalates (LMWP) (Wormuth et al. 2006). Phthalates and BPA have a short biological half-life (i.e., few hours or days), but their ubiquity implies a constant but highly variable exposure (Braun et al. 2012).

BPA and phthalates and their metabolites have known endocrine-disrupting properties that may disrupt hormonal balance even at low doses of exposure (Casals-Casas and Desvergne 2011). BPA and phthalate metabolites can interact with the estrogen, androgen, thyroid hormone, glucocorticoid, and/or peroxisome proliferator–activated receptors (PPARs) that regulate important biological processes for the control of adipogenesis, insulin levels, fluid retention, and bone metabolism (Ahmadian et al. 2013; Casals-Casas and Desvergne 2011). Some of these hypothesized effects, especially those mediated by the steroid hormone receptors, could be sex-specific. In animal studies, effects of prenatal exposure to BPA and phthalate metabolites are controversial, with studies reporting a reduction, gain, or no effects on body weight (Kim et al. 2001; Rubin et al. 2001; Tanaka 2005) and studies reporting increased femur length, skeletal malformations, or retardation in the ossification (Agas et al. 2013; Kim et al. 2001). Results from human epidemiological studies are also inconsistent (Chou et al. 2011; Huang et al. 2014; Lee et al. 2008, 2014; Miao et al. 2011; Philippat et al. 2012, 2014; Snijder et al. 2013; Suzuki et al. 2010; Wolff et al. 2008; Zhang et al. 2009). Most of the epidemiological studies have measured child anthropometric parameters at birth (Chou et al. 2011; Huang et al. 2014; Lee et al. 2014; Miao et al. 2011; Philippat et al. 2012; Suzuki et al. 2010; Wolff et al. 2008; Zhang et al. 2009). Only three studies assessed fetal growth characteristics associated with prenatal BPA exposure (Lee et al. 2008; Philippat et al. 2014; Snijder et al. 2013); none did so for phthalates. The majority of studies have determined BPA and phthalate metabolites in urine or blood in one spot sample collected during pregnancy; only one study measured BPA in more than one urine sample and found that the exposure–response relationship became progressively attenuated when fewer measurements were available (Snijder et al. 2013). This emphasizes the necessity of using multiple measurements per subject to obtain a more reliable measurement of exposure levels. Furthermore, data from *in vivo* studies have revealed sex-dependent effects on body weight in rodents exposed perinatally to BPA (Rubin and Soto 2009), but few human studies have been able to evaluate sex-specific effects and findings are still controversial (Chou et al. 2011; Huang et al. 2014; Lee et al. 2014).

In this study we assessed whether prenatal exposure to BPA and phthalates may influence fetal growth and birth outcomes in a Spanish birth cohort of 488 mother–child pairs.

## Methods

*Study population.* The INMA study (INfancia y Medio Ambiente; Childhood and Environment) is a population-based birth cohort study that recruited 657 pregnant women in the Spanish region of Sabadell between 2004 and 2006 (Guxens et al. 2012). Women were recruited at their first routine prenatal care visit (mean ± SD = 13.4 ± 1.7 weeks of gestation) in the primary care center if they fulfilled the inclusion criteria: age ≥ 16 years, intention to deliver in the reference hospital, singleton pregnancy, unassisted conception, and no communication problems (Guxens et al. 2012). The study was approved by the Ethics Committee of the reference hospital, and all participants gave their written informed consent.

*Prenatal BPA and phthalate exposure.* Maternal urine samples were collected at 12 ± 1.7 and 32 ± 1.4 weeks of gestation and stored in polypropylene (for BPA analysis) or polyethylene tubes (for phthalate metabolites analysis) at –20°C. Total BPA (free plus conjugated) was quantified by liquid chromatography–mass spectrometry in the Department of Analytical Chemistry laboratory–University of Cordoba (Spain) (Casas et al. 2013). Eight phthalate metabolites (free plus conjugated) were determined by liquid chromatography–mass spectrometry in the Bioanalysis Research Group at the Hospital del Mar Medical Research Institute (Spain) (Valvi et al. 2015b). Limits of detection (LODs) for each analyte are listed in Table 1. Creatinine was determined at the Echevarne laboratory of Barcelona (Spain) by the Jaffé method (kinetic with target measurement, compensated method) with Beckman Coulter^©^ reactive in AU5400 (IZASA®). All BPA and phthalate metabolites concentrations were divided by urinary creatinine concentrations to control for urine dilution. Selection of women for BPA and phthalates measurements was based on criteria set in previous studies conducted in this population (Casas et al. 2013; Valvi et al. 2015b): BPA concentrations were measured in 479 mothers, whereas phthalate metabolites were measured in 391 mothers (see Supplemental Material, Figure S1).

**Table 1:** Maternal urinary concentrations of creatinine, BPA,*^a^* and phthalate*^a^* metabolites.

Compounds	*n*	LOD (μg/L)	% < LOD (1st–3rd trimester)	GM (95% CI)	Minimum–maximum	ICC^*b*^ (95% CI)
Creatinine (g/L)
1st trimester	488			0.82 (0.78, 0.86)	0.1–2.7	0.22 (0.14, 0.31)
3rd trimester	488			0.88 (0.83, 0.92)	0.1–3.0
Unadjusted (μg/L)
BPA	470	0.1	0–0.6	2.3 (2.1, 2.4)	0.3–61.8	0.15 (0.06, 0.24)
Phthalates
ΣDEHPm	390			92.5 (86.5, 99.1)	14.3–1428.9
MEHP	390	1	0.5–0.8	9.6 (9.0, 10.3)	1.3–202.0	0.20 (0.10, 0.29)
MEHHP	390	0.5	0	25.5 (23.6, 27.5)	3.2–536.0	0.08 (0.00, 0.18)
MEOHP	390	0.5	0	19.0 (17.7, 20.4)	2.3–342.8	0.08 (0.00, 0.18)
MECPP	390	1	0–0.3	36.2 (33.7, 38.9)	4.6–476.1
MBzP	390	0.5	0.8	11.1 (10.2, 12.1)	0.8–336.4	0.22 (0.12, 0.31)
ΣLMWPm	390			428.7 (392.9, 467.8)	43.1–5183.1
MEP	390	1	0–0.3	335.6 (303.4, 371.3)	21.9–5115.1	0.23 (0.14, 0.33)
MiBP	390	0.5	0	28.8 (26.7, 31.0)	4.0–367.6	0.22 (0.13, 0.32)
MnBP	390	1	0.8	29.0 (26.7, 31.5)	3.4–402.6	0.20 (0.10, 0.29)
Creatinine-adjusted (μg/g)
BPA	470	0.1	0–0.6	2.6 (2.4, 2.8)	0.3–69.4	0.14 (0.05, 0.22)
Phthalates
ΣDEHPm	390			106.1 (99.8, 112.8)	26.5–1670.0
MEHP	390	1	0.5–0.8	11.3 (10.6, 12.1)	1.8–266.9	0.18 (0.08, 0.28)
MEHHP	390	0.5	0	29.0 (27.1, 31.0)	5.3–503.4	0.06 (0.00, 0.16)
MEOHP	390	0.5	0	21.7 (20.3, 23.1)	4.1–378.3	0.06 (0.00, 0.16)
MECPP	390	1	0–0.3	41.4 (38.9, 44.1)	7.7–718.9	0.19 (0.09, 0.29)
MBzP	390	0.5	0.8	12.6 (11.6, 13.6)	1.5–405.1	0.23 (0.14, 0.33)
ΣLMWPm	390			494.8 (456.2, 536.7)	65.2–10030.2
MEP	390	1	0–0.3	389.1 (353.4, 428.4)	34.0–9379.8	0.23 (0.13, 0.32)
MiBP	390	0.5	0	33.0 (31.0, 35.1)	5.1–334.2	0.19 (0.09, 0.29)
MnBP	390	1	0.8	32.7 (30.4, 35.2)	5.8–835.7	0.19 (0.09, 0.29)
Abbreviations: BPA, bisphenol A; CI, confidence interval; DEHPm, di-2-ethylhexyl phthalate metabolites; GM, geometric mean; ICC, intraclass correlation coefficient; LMWPm, low molecular weight phthalate metabolites; LOD, limit of detection; MBzP, mono-benzyl phthalate; MECPP, mono(2-ethyl-5-carboxy-pentyl) phthalate; MEHHP, mono(2-ethyl-5-hydroxy-hexyl) phthalate; MEHP, mono(2-ethyl-hexyl) phthalate; MEOHP, mono(2-ethyl-5-oxo-hexyl) phthalate; MEP, mono-ethyl phthalate; MiBP, mono-isobutyl phthalate; MnBP, mono-*n*-butyl phthalate. ^***a***^Average of measurements at two time points in the first and third trimesters of pregnancy. ^***b***^The ICC is calculated by dividing the between-person variability by the sum of the between- and within-person variability. Values range from 0 (i.e., no reproducibility of the same measurement within a subject) to 1 (i.e., perfect reproducibility).						

*Fetal growth and birth outcomes.* Routine fetal ultrasound examinations, regardless of study participation, were scheduled at 12, 20, and 34 weeks of pregnancy by obstetricians who specialized in conducting this type of examinations at the respective hospitals. The fetal parameters recorded (millimeters) were femur length (FL), head circumference (HC), abdominal circumference (AC), and biparietal diameter (BPD). Estimated fetal weight (EFW) was calculated using the Hadlock algorithm (Hadlock et al. 1985). We had access to the records of any other ultrasound performed to women in the same hospital unit during their pregnancy, including an early ultrasound (< 10 weeks of pregnancy). In this first ultrasound, crown–rump length (CRL) was measured and used to establish gestational age only when the difference between CRL and the self-reported last menstrual period was ≥ 7 days. Birth weight (grams) was recorded by trainee midwifes at delivery, whereas birth length and head circumference (both measured in centimeters but expressed in millimeters in the present analysis) were measured by a nurse when the neonate arrived at the hospital ward within the first 12 hr of life. We also collected placentas at the time of delivery and analyzed placenta weight as a measure of growth *in utero*. Placentas were examined and weighed without deciduas basalis and chorionic plate. In our analysis we included women with at least two ultrasound measurements conducted at any time during pregnancy and with at least one birth outcome available at the time of delivery (see Supplemental Material, Figure S1).

Linear mixed models were used to obtain longitudinal growth curves for the five fetal parameters (see Supplemental Material, Figure S2). A detailed description of these growth curves is provided by Iñiguez et al. (2015). To obtain an individualized rather than a population-based growth standard, we tested physiological factors known to affect fetal growth (Mamelle et al. 2006) and their interactions with gestational age in days using the likelihood ratio (LR) test (*p* < 0.05) through a forward selection procedure. These physiological factors included maternal age, parity, country of origin, maternal height, maternal prepregnancy weight or body mass index (BMI), paternal height, weight or BMI, and child sex. Only the significant ones were included in the models (see Supplemental Material, Table S1). Growth models were then applied to calculated standard deviation (SD) scores at 12, 20, and 34 weeks of gestation, representing cross-sectional estimates of fetal size (e.g., size at 12 weeks), and SD scores over the week intervals 12–20 and 20–34, representing fetal growth between two periods (e.g., growth between 12 and 20 weeks) (Gurrin et al. 2001). Growth curves were generated by using R software (R Core Team 2013). Because birth outcomes depend largely on the gestational age, growth curves for birth weight, length, head circumference, and placental weight were fitted to further standardize them to week 40 of gestation using the Box–Cox power exponential method (Rigby and Stasinopoulos 2004) and adjusting by sex.

*Covariates.* Information on maternal age, education, social class, country of origin, weight and height, parity, health history, marital status, dietary intake, use of household cleaning products, active smoking, time of urine collection, and season of birth was obtained by self-reported questionnaires administrated by trained interviewers at weeks 12 and 32 of pregnancy. Child sex was obtained from clinical records. Cotinine was determined in urine at 32 pregnancy weeks, using commercial competitive enzyme immunoassay microplate test kits (OraSure Technologies Inc., Bio-Rad) with a limit of quantification (LOQ) of 4.0 μg/L.

*Statistical analysis.* Urinary concentrations of BPA and phthalate metabolites below the LOD were assigned a value of half the LOD. Reproducibility of urinary BPA and phthalate metabolites between the first and third trimesters was obtained by calculating the intraclass correlation coefficient (ICC) using random one-way intercept linear mixed models. We also calculated Pearson correlations of the log-transformed compounds concentrations. Because of the short biological half-lives of these compounds (Koch and Calafat 2009), we used the average of the creatinine-adjusted concentrations determined in 12 and 32 weeks of pregnancy to provide a better estimation of exposure during pregnancy. Because of the approximate log-normal distribution of urinary concentrations, average creatinine-adjusted concentrations of pollutants were log_2_-transformed.

Generalized additive models were used to evaluate linearity of our exposure–outcome relationships. Associations between BPA/phthalates and SD scores for fetal size/growth, and birth parameters were then examined by univariate and multivariate linear regression models. To determine the covariates included in the multivariate models, we applied directed acyclic graphs (DAGs) (Shrier and Platt 2008). Covariates were included in the DAGs if they were described to be associated with the exposure or the outcome in previous literature (Aguilera et al. 2010; Casas et al. 2013; Snijder et al. 2013; Valvi et al. 2013), and such associations were shown in bivariate analyses of our data (see Supplemental Material, Table S2) (*p* ≤ 0.1). Based on the DAGs, the final multivariate models were adjusted for maternal education (primary, secondary, university), smoking during pregnancy (never smoked, not during pregnancy, during pregnancy), and parity (nulliparous, multiparous). Departing from this multivariate model, we then conducted a forward stepwise selection procedure including other potential confounders and ancestors and testing if they changed the coefficient by > 10%: birth season, urinary cotinine levels during pregnancy, consumption of canned fish during pregnancy, time of urine collection, and use of household cleaning products. Birth season (winter, spring, summer, autumn) and urinary cotinine levels during pregnancy [non–secondhand tobacco smoke (SHS) exposure, < 18 ng/mL; SHS exposed, 18–50 ng/mL; active smoker, > 50 ng/mL] changed most of the coefficients by > 10% and hence they were included in all final models. Because fetal HC growth models were already adjusted for parity (see Supplemental Material, Table S1), this variable was not included in the fetal HC multivariate models. Finally, newborn’s HC models were also adjusted for type of delivery (vaginal, instrumental, cesarean) because passage through the birth canal may influence HC at birth. We are only presenting the adjusted models because they yielded results similar to the unadjusted ones.

Results for phthalates are presented for the sum (micrograms per liter) of the four DEHP [di(2-ethylhexyl) phthalate] metabolites {ΣDEHPm: MEHP [mono(2-ethylhexyl) phthalate], MEHHP [mono(2-ethyl-5-hydroxyhexyl) phthalate], MEOHP [mono(2-ethyl-5-oxohexyl) phthalate], and MECPP [mono(2-ethyl-5-carboxypentyl) phthalate]}, MBzP (monobenzyl phthalate), the sum (micrograms per liter) of the three LMWP metabolites [ΣLMWPm: MEP (monoethyl phthalate), MiBP (monoisobutyl phthalate), and MnBP (mono-*n*-butyl phthalate)], and for each single phthalate metabolite. Because these parent and metabolites compounds are hormonally active, we report all primary analyses stratified by sex, in addition to reporting estimated for boys and girls combined. We performed likelihood ratio tests of interaction terms for exposure and sex to identify significant modification by sex based on *p* ≤ 0.1. To evaluate the robustness of our results, we conducted various sensitivity analyses and considered whether the findings were generally consistent throughout the different models. First, we repeated the models adjusting for urinary creatinine concentrations as has been performed in other studies (Philippat et al. 2012). Second, we also repeated the analyses excluding extreme values of creatinine that ranged between < 0.3 g/L and > 3.0 g/L (*n* = 40) to prevent “dilution effects” in the urine samples (Aitio 1996). Third, we performed multi-pollutant models where the four main exposure variables were included (BPA, ΣDEHPm, MBzP, and ΣLMWPm) and evaluated whether coefficient changed by comparing results with the single-pollutant model after restricting the data to observations with complete data for all four pollutants. All statistical analyses were conducted with Stata 12.0 statistical software (StataCorp, College Station, TX, USA).

## Results

*Study population and exposure characteristics.* Of the 657 pregnant women initially enrolled, 488 mother–child pairs had information on prenatal BPA (*n* = 470) and/or phthalate metabolites concentrations (*n* = 390) and fetal growth parameters or birth outcomes (see Supplemental Material, Figure S1). Women included in the study were more likely to be Spanish, well-educated, and from a higher socioeconomic position than INMA-Sabadell participants not included in the analysis (data not shown). Complete details of the characteristics of the study population are given in Supplemental Material, Table S2.

BPA and phthalate metabolites were detected in most of the urine samples (0–0.8% < LOD) (Table 1). The unadjusted geometric mean (GM) BPA concentration was 2.3 μg/L [95% confidence interval (CI): 2.1, 2.4]. Among phthalate metabolites, the low molecular weight phthalate MEP presented the highest concentration (GM: 335.6 μg/L; 95% CI: 303.4, 371.3), whereas MEHP and MBzP showed the lowest (GM: 9.6 μg/L; 95% CI: 9.0, 10.3; GM: 11.1 μg/L; 95% CI: 10.2, 12.1, respectively). The ICCs comparing samples collected in the first and third trimesters ranged from 0.06 to 0.23, with the highest ICC for MBzP and MEP and the lowest for MEHHP and MEOHP (Table 1). Low correlations were found between the different exposure groups (BPA, ΣDEHPm, MBzP, and ΣLMWPm), with Pearson correlation coefficients ranging from 0.14 to 0.32 (see Supplemental Material, Table S3). A total of 1,452 ultrasound examinations were performed for the 488 pregnancies. Most women (*n* = 473) had one routine ultrasound examination in each trimester of pregnancy but 15 (3%) women had four to six examinations. The mean (± SD) gestational age, birth and placental weight were 39.7 ± 1.4 weeks, 3,319 ± 391 g, and 602 ± 110 g, respectively (data not shown).

**Figure 1:**
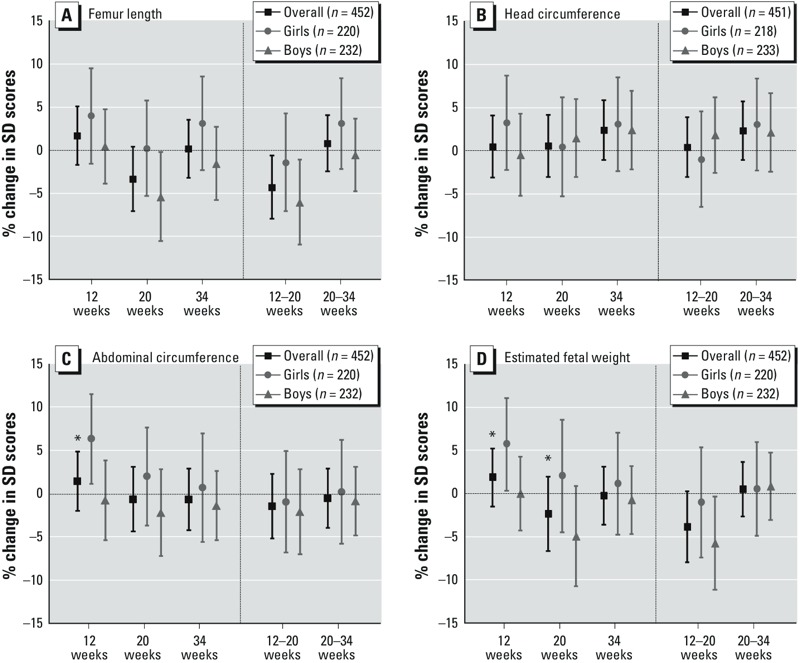
Adjusted associations between maternal urinary BPA levels (μg/g creatinine) and fetal size and growth parameters in the overall population, in girls, and in boys: femur length (*A*), head circumference (*B*), abdominal circumference (*C*), and estimated fetal weight (*D*). BPA, bisphenol A. Mean percent difference in standard deviation scores per doubling of BPA levels (levels were log_2_-transformed). Femur length model was adjusted for maternal age, maternal height, paternal weight, maternal country of origin, maternal education, smoking during pregnancy, parity, birth season, and urinary cotinine levels during pregnancy. Head circumference model was adjusted for maternal age, maternal height/weight, paternal weight, maternal education, smoking during pregnancy, parity, birth season, and urinary cotinine levels during pregnancy. Abdominal circumference model was adjusted for maternal age, maternal height/weight, paternal weight, maternal country of origin, maternal education, smoking during pregnancy, parity, birth season, and urinary cotinine levels during pregnancy. Estimated fetal weight model was adjusted for maternal age, maternal height, paternal weight, maternal country of origin, maternal education, smoking during pregnancy, parity, birth season, and urinary cotinine levels during pregnancy. *p-Interaction for sex ≤ 0.1.

*BPA and fetal growth and birth outcomes.* Exposure to BPA during pregnancy was negatively associated with FL and EFW from 12 to 20 weeks resulting in a percent reduction in SD scores of –4.26% (95% CI: –7.91, –0.65%) and –3.84% (95% CI: –7.93, 0.30%), respectively, for each doubling of concentration of maternal urinary BPA (Figure 1). Negative associations of BPA with growth in FL and EFW from 12 to 20 weeks were evident in boys but not in girls (e.g., for growth in EFW, –5.74%; 95% CI: –11.08, –0.29% and –0.98%; 95% CI: –7.35, 5.42% in boys and girls, respectively), but the differences between boys and girls were not significant (*p* interaction = 0.22 for FL, *p* = 0.15 for EFW) (Figure 1). Significant differences by sex in EFW at 12 weeks and 20 weeks were due to a significant positive association with size at 12 weeks in girls and a null association in boys (*p* interaction = 0.05), whereas at 20 weeks there was a weak positive association with EFW in girls and a stronger nonsignificant negative association in boys (*p* interaction = 0.05) (Figure 1). There was also a significant difference by sex in the association of BPA with AC at 12 weeks, with a positive association among girls (6.41%; 95% CI: 1.16, 11.54%) and no association among boys (–0.75%; 95% CI: –5.35, 3.87%) (*p* interaction = 0.03) (Figure 1). Prenatal BPA exposure was not significantly associated with any of the birth outcomes studied, though there was a small and nonsignificant association with gestational age at birth (1.19 weeks per doubling of BPA concentration; 95% CI: –0.19, 2.58) (Table 2). None of the associations that were statistically significant for BPA in the main analysis (for FL and EFW growth from 12 to 20 weeks, and for EFW and AC at 12 weeks in girls) were statistically significant in all the sensitivity analyses related to creatinine (i.e., adjusted for creatinine or after excluding samples with extreme creatinine values) (see Supplemental Material Table S4). The inclusion of all four main exposures in one multi-pollutant model led to changes in coefficients between 5 and 80% compared with results with the single-pollutant model (see Supplemental Material, Table S5).

**Table 2:** Adjusted associations between maternal urinary BPA and phthalate metabolites levels (μg/g creatinine) and birth outcomes in the overall population, in girls, and in boys.

Compounds	Overall		Girls		Boys		*p* Interaction
	*n*	β (95% CI)	*n*	β (95% CI)	*n*	β (95% CI)	
BPA
Weight (g)	448	–16.24 (–54.10, 21.62)	219	–21.56 (–82.93, 39.82)	229	–11.01 (–57.39, 35.37)	0.99
Placental weight (g)	398	0.53 (–10.79, 11.86)	193	–8.01 (–28.03, 12.01)	205	6.98 (–6.47, 20.44)	0.28
Length (mm)	441	–0.37 (–2.00, 1.25)	212	–0.64 (–3.21, 1.93)	229	–0.27 (–2.31, 1.77)	0.93
Head circumference (mm)	435	0.47 (–0.66, 1.60)	208	0.38 (–1.47, 2.23)	227	0.54 (–0.77, 1.85)	0.77
Gestational age (weeks)	453	1.19 (–0.19, 2.58)	220	1.65 (–0.56, 0.39)	233	0.63 (–1.21, 2.46)	0.54
ΣDEHPm
Weight (g)	371	15.56 (–28.75, 59.87)	179	5.92 (–60.59, 72.42)	192	14.68 (–44.11, 73.48)	0.82
Placental weight (g)	325	1.31 (–12.30, 14.93)	154	–14.25 (–36.73, 8.24)	171	13.94 (–2.85, 30.72)	0.04
Length (mm)	364	0.38 (–1.59, 2.35)	172	0.78 (–2.06, 3.61)	192	–0.25 (–2.91, 2.40)	0.84
Head circumference (mm)	359	0.16 (–1.15, 1.47)	169	0.00 (–1.98, 1.97)	190	0.17 (–1.46, 1.80)	0.56
Gestational age (weeks)	375	–0.13 (–1.72, 1.46)	180	0.20 (–2.23, 2.63)	195	–0.48 (–2.65, 1.69)	0.71
MBzP
Weight (g)	371	14.11 (–19.08, 47.29)	179	–27.30 (–79.41, 24.80)	192	47.78 (5.78, 89.78)	0.04
Placental weight (g)	325	0.75 (–9.79, 11.30)	154	–20.57 (–39.22, –1.92)	171	12.53 (0.27, 24.79)	0.02
Length (mm)	364	–0.42 (–1.90, 1.07)	172	–1.42 (–3.67, 0.84)	192	0.43 (–1.49, 2.35)	0.24
Head circumference (mm)	359	–0.07 (–1.05, 0.92)	169	–0.92 (–2.47, 0.62)	190	0.70 (–0.48, 1.88)	0.07
Gestational age (weeks)	375	–0.39 (–1.61, 0.83)	180	–0.43 (–2.34, 1.48)	195	–0.43 (–2.06, 1.20)	0.97
ΣLMWPm
Weight (g)	371	–10.02 (–42.84, 22.80)	179	–11.55 (–62.08, 38.97)	192	6.16 (–37.64, 49.95)	0.47
Placental weight (g)	325	4.20 (–6.05, 14.46)	154	–1.65 (–19.31, 16.01)	171	9.18 (–3.42, 21.78)	0.18
Length (mm)	364	–0.74 (–2.20, 0.72)	172	–0.24 (–2.39, 1.92)	192	–0.77 (–2.75, 1.20)	0.97
Head circumference (mm)	359	–0.54 (–1.51, 0.43)	169	–0.13 (–1.62, 1.36)	190	–0.62 (–1.85, 0.61)	0.89
Gestational age (weeks)	375	0.27 (–0.94, 1.48)	180	0.38 (–1.48, 2.24)	195	–0.29 (–1.96, 1.39)	0.42
Abbreviations: BPA, bisphenol A; CI, confidence interval; DEHPm, di(2-ethylhexyl) phthalate metabolites; MBzP, mono-benzyl phthalate; LMWPm, low-molecular-weight phthalate metabolites. Betas represent the estimated difference in each outcome associated per doubling of exposure levels (levels were log_2_-transformed). All models were adjusted for maternal education, smoking during pregnancy, parity, birth season, and urinary cotinine levels during pregnancy. Head circumference models also adjusted for type of delivery.							

**Figure 2:**
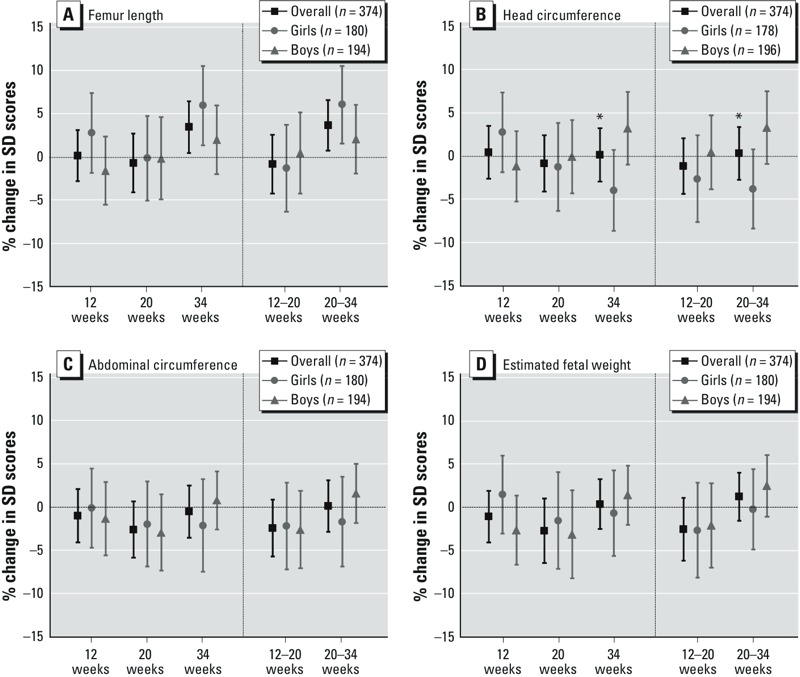
Adjusted associations between maternal urinary MBzP levels (μg/g creatinine) and fetal size and growth parameters in the overall population, in girls, and in boys: femur length (*A*), head circumference (*B*), abdominal circumference (*C*), and estimated fetal weight (*D*). MBzP, mono-benzyl phthalate. Mean percent difference in standard deviation scores per doubling of MBzP levels (levels were log_2_-transformed). Femur length model was adjusted for maternal age, maternal height, paternal weight, maternal country of origin, maternal education, smoking during pregnancy, parity, birth season, and urinary cotinine levels during pregnancy. Head circumference model was adjusted for maternal age, maternal height/weight, paternal weight, maternal education, smoking during pregnancy, parity, birth season, and urinary cotinine levels during pregnancy. Abdominal circumference model was adjusted for maternal age, maternal height/weight, paternal weight, maternal country of origin, maternal education, smoking during pregnancy, parity, birth season, and urinary cotinine levels during pregnancy. Estimated fetal weight model was adjusted for maternal age, maternal height, paternal weight, maternal country of origin, maternal education, smoking during pregnancy, parity, birth season, and urinary cotinine levels during pregnancy. *p interaction for sex ≤ 0.1.

*Phthalate metabolites and fetal growth and birth outcomes.* Exposure to ΣDEHPm and its single metabolites during pregnancy was not significantly associated with any of the fetal growth (see Supplemental Material, Table S6) or birth outcomes assessed (Table 2; see also Supplemental Material, Table S7). A doubling of prenatal MBzP exposure was associated with a 3.70% increase (95% CI: 0.75, 6.63%) in FL growth from 20 to 34 weeks (Figure 2; see also Supplemental Material, Table S8). Among girls a doubling of MBzP was associated with a 6.12% higher FL growth from 20 to 34 weeks (95% CI: 1.61, 10.55%) compared with 2.10% (95% CI: –1.86, 6.04%) in boys, though the difference was not significant (*p* interaction = 0.15) (Figure 2). At birth, a doubling of MBzP was associated with higher birth weight in boys (48 g; 95% CI: 6, 90) but not in girls (–27 g; 95% CI: –79, 25) (*p* interaction = 0.04), and was positively associated with placental weight in boys (13 g; 95% CI = 0.3, 25) but negatively associated in girls (–21 g; 95% CI: –39, –2) (*p* interaction = 0.02) (Table 2). After conducting various sensitivity analyses related to creatinine (i.e., adjusted for creatinine, excluding creatinine extreme values) the association between MBzP and placental weight in girls and boys was not statistically significant (see Supplemental Material, Table S4). Associations between MzBP and FL and birth weight were consistent after including the four main exposure variables (see Supplemental Material, Table S5).

**Figure 3:**
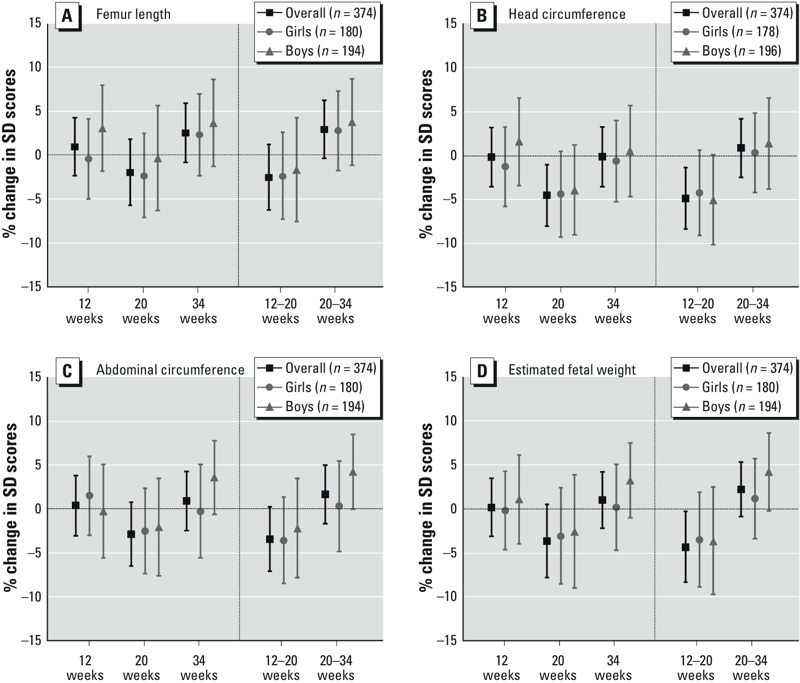
Adjusted associations between maternal urinary MnBP levels (μg/g creatinine) and fetal and size growth parameters in the overall population, in girls, and in boys: femur length (*A*), head circumference (*B*), abdominal circumference (*C*), and estimated fetal weight (*D*). MnBP, mono-*n*-butyl phthalate. Mean percent difference in standard deviation scores per doubling of MnBP levels (levels were log_2_-transformed). Femur length model was adjusted for maternal age, maternal height, paternal weight, maternal country of origin, maternal education, smoking during pregnancy, parity, birth season, and urinary cotinine levels during pregnancy. Head circumference model was adjusted for maternal age, maternal height/weight, paternal weight, maternal education, smoking during pregnancy, parity, birth season, and urinary cotinine levels during pregnancy. Abdominal circumference model was adjusted for maternal age, maternal height/weight, paternal weight, maternal country of origin, maternal education, smoking during pregnancy, parity, birth season, and urinary cotinine levels during pregnancy. Estimated fetal weight model was adjusted for maternal age, maternal height, paternal weight, maternal country of origin, maternal education, smoking during pregnancy, parity, birth season, and urinary cotinine levels during pregnancy.

Exposure to ΣLMWPm was not significantly associated with any of the fetal size or fetal growth outcomes (see Supplemental Material, Table S8) or any of the birth outcomes assessed (Table 2; see also Supplemental Material, Table S7). The metabolite MnBP was associated with significantly lower HC growth from 12 to 20 weeks (–4.88%; 95% CI: –8.36, –1.36%) and EFW growth from 12 to 20 weeks (–4.32%; 95% CI: –8.33, –0.27%) (Figure 3; see also Supplemental Material, Table S8). MnBP was associated with greater AC and EFW growth from 20 to 34 weeks in boys (4.29%; 95% CI: 0.01, 8.53% and 4.27%; 95% CI: –0.18, 8.68%, respectively) but not in girls (0.39%; 95% CI: –4.76, 5.54% and 1.22%; 95% CI: –3.36, 5.78%, respectively), though differences were not significant (*p* interaction = 0.21 and 0.31, respectively) (Figure 3). MnBP was also associated with higher birth weight in boys (57 g; 95% CI: 3, 110) but not girls (11 g; 95% CI: –40, 62) (*p* interaction = 0.29) (see Supplemental Material, Table S7). Further, the metabolite MiBP was associated with lower birth weight in girls (–73 g; 95% CI: –137, –9) but not among boys (19 g; 95% CI: –35, 74) (*p* interaction = 0.08) (see Supplemental Material, Table S6). Associations of individual LMWP metabolites with other birth outcomes were not statistically significant overall or in girls or boys (see Supplemental Material, Table S7). Of the associations that were statistically significant in primary analyses, only the association between MnBP and HC growth from 12 to 20 weeks met our criteria for consistency (see Supplemental Material, Tables S4 and S5).

## Discussion

In this population of pregnant women with a common exposure to BPA and phthalates, we found few associations between these compounds and fetal growth and birth outcomes. The HMWPm MBzP was significantly associated with greater FL growth from 20 to 34 weeks in the overall population and with significantly higher birth weight in boys, but not in girls. In contrast, the LMWPm MnBP was associated with lower HC growth from 12 to 20 weeks in the overall population, with no evidence of sex differences. These associations persisted after sensitivity analyses, including adjustment for other pollutants. Maternal urinary BPA and DEHPm concentrations were not significantly associated with any of the growth outcomes assessed either during fetal life or at birth.

Concentrations of BPA among pregnant women in our population were similar to populations in other studies where BPA and fetal growth outcomes were evaluated (Lee et al. 2008; Philippat et al. 2014; Snijder et al. 2013). In two of these studies, high prenatal BPA concentrations were associated with reduced fetal growth; but the sample size was relatively small [*n* = 125 in Lee et al. (2008); *n* = 80 in Snijder et al. (2013)]. Also, they did not assess either sex-specific differences or the influence of other pollutants. In the EDEN cohort study, which included only boys, no associations were observed between BPA and fetal growth (Philippat et al. 2014). In our population, although we found statistically significant sex-specific associations between prenatal BPA exposure and FL, AC, and EFW, these associations did not meet our criteria for robustness in sensitivity analyses. We also did not find significant associations between BPA and any of the anthropometric measures at birth. Epidemiological studies assessing birth outcomes have also shown contradictory results, with some studies showing an increase in anthropometric measures (Lee et al. 2014; Philippat et al. 2012), and others finding a decrease (Chou et al. 2011; Miao et al. 2011), or null associations (Wolff et al. 2008). In animal studies discrepant results also exist regarding BPA effects on fetal weight gain and bone development (Agas et al. 2013; Kim et al. 2001; Somm et al. 2009). It is worth noting that prenatal BPA exposure was positively associated with waist circumference and BMI at 4 years in the same INMA-Sabadell birth cohort, but not at earlier ages (Valvi et al. 2013). Thus, whether prenatal exposure to BPA may start affecting fetal growth during pregnancy but with effects to be observed only at later ages needs to be further explored (Unüvar and Büyükgebiz 2012).

Phthalate metabolite levels in urine of pregnant women from our population were of similar magnitude to those reported in other studies assessing phthalates in relation to birth outcomes (Philippat et al. 2012; Suzuki et al. 2010; Wolff et al. 2008). Two other studies measured the parent and not the metabolite phthalate compounds in blood, so the comparison of levels with those of the present study is difficult (Huang et al. 2014; Zhang et al. 2009). Again, inconsistent results were found between studies, with one showing an increase in length and head circumference at birth (Wolff et al. 2008); some showing a decrease in birth weight, length, and head circumference among other growth parameters (Huang et al. 2014; Zhang et al. 2009); and others reporting no associations with any phthalates exposure (Philippat et al. 2012; Suzuki et al. 2010). To our knowledge, no study thus far has evaluated ultrasound measurements in relation to phthalates exposure during pregnancy. We found that prenatal exposure to the HMW phthalate MBzP was positively associated with fetal FL growth from 20 to 34 weeks in the overall population and with higher birth weight in boys but not among girls. Our results are in line with toxicological and animal studies suggesting that exposure to MBzP or its parent compound benzyl butyl phthalate (BBP) may stimulate adipogenesis and increase osteoblast proliferation (Agas et al. 2013; Hurst and Waxman 2003). On the contrary, exposure to the LMWPm MnBP seemed to be associated with a decrease in fetal HC early in pregnancy. Huang et al. (2014) observed a reduction in HC and in other fetal parameters at birth associated with exposure to dibutyl phthalate (DBP), the precursor of MnBP. Zhang et al. (2009) assessed birth weight and found an increased risk of low birth weight associated with DBP exposure; however, both of these studies measured phthalates exposure in blood samples, which may not produce reliable estimates of exposure. In rodents, decreased growth was also observed after exposure to DBP (Marsman 1995). In the INMA-Sabadell birth cohort, boys presented reduced weight gain and reduced risk of overweight from birth until 7 years of age linked to a high HMWPm exposure during pregnancy; no associations were found for LMWPm (Valvi et al. 2015a). Children with higher weight at birth compared with children with lower birth weights may tend to grow more slowly during the first years of life, and they could be at lower risk of obesity later in childhood and adult life (Labayen et al. 2012). More studies are needed to disentangle the potential effects of exposure to phthalates during pregnancy on fetus and child growth.

This study has some limitations. First, although we used the average of two BPA and phthalate measurements during pregnancy, the high within-person and within- and between-day variability of these compounds means that exposure misclassification cannot be ruled out. Such misclassification is likely to be random with respect to our outcomes, and is thus most likely to have led to an attenuation of associations (Pollack et al. 2013). Second, we were able to include BPA and phthalate exposures in one model, and we showed that those more strongly associated to fetal growth and birth outcomes were MBzP and MnBP. Third, because of the small number of ultrasounds performed from week 35 onward, we could not assess the influence of BPA and phthalates on fetal growth during late pregnancy (i.e., weeks 35–38), when most of the constitutional variation in fetal parameters occur (Hindmarsh et al. 2002). Fourth, INMA-Sabadell cohort participants included in the analysis were more likely to be Spanish, more educated, and from a higher socioeconomic class than those excluded from the analysis. Lower education and lower socioeconomic class are linked to higher urine concentrations of BPA and phthalates in our population (Casas et al. 2013; Valvi et al. 2015b) and to higher risk of adverse pregnancy outcomes (de Graaf et al. 2013); thus, the most highly exposed and most susceptible women could have been excluded from analysis. Finally, we performed quite a large number of comparisons between exposures and outcomes, which may have led to spurious findings.

The major strength of this paper relies on its prospective design and the use of repeated measurements of fetal biometry. Also, we had repeated measurements of BPA and phthalates at the first and third trimester of pregnancy in almost 500 pregnant women, making this, to our knowledge, the largest and most extensive study on this topic. Finally, we had data available for a large number of potential confounders.

## Conclusions

This study is one of the first to combine repeat exposure biomarker measurements and multiple growth measures during pregnancy. We did not find consistent or strong evidence of associations between BPA or phthalate exposures and fetal growth, though the phthalate metabolites MBzP and MnBP were associated with some fetal growth parameters. These findings require replication. Production of some phthalates and BPA has already been banned in some countries and replaced by other chemicals such as the BPA analogs BPE or BPF that can also have endocrine-disrupting activity (Rosenmai et al. 2014). Investigating the effects of phthalates and BPA is relevant for improving current knowledge of health effects in children and provides further guidelines for an effective regulatory policy, given that they have similar structure and mechanisms of action.

## Supplemental Material

(672 KB) PDF
