# Supplementary material for: Exposure to Bisphenol A and Phthalates during Pregnancy and Ultrasound Measures of Fetal Growth in the INMA-Sabadell Cohort
Source: Environ Health Perspect. 2015 Jul 21;124(4):521–8. doi: 10.1289/ehp.1409190 (PMC4829997; doi:10.1289/ehp.1409190)
Supplement: (672 KB) PDF [file ehp.1409190.s001.acco.pdf]

Environ Health Perspect
DOI: 10.1289/ehp.1409190

Note to Readers: *EHP* strives to ensure that all journal content is accessible to all readers. However, some figures and Supplemental Material published in *EHP* articles may not conform to 508 standards due to the complexity of the information being presented. If you need assistance accessing journal content, please contact ehp508@niehs.nih.gov. Our staff will work with you to assess and meet your accessibility needs within 3 working days.

Supplemental Material

Exposure to Bisphenol A and Phthalates during Pregnancy and Ultrasound Measures of Fetal Growth in the INMA-Sabadell Cohort

Maribel Casas, Damaskini Valvi, Ana Ballesteros-Gomez, Mireia Gascon, Mariana F. Fernández, Raquel Garcia-Esteban, Carmen Iñiguez, David Martínez, Mario Murcia, Nuria Monfort, Noelia Luque, Soledad Rubio, Rosa Ventura, Jordi Sunyer, and Martine Vrijheid

Table of Contents

Figure S1. Flowchart of the study population
Figure S2. Fetal growth curves for femur length, head circumference, abdominal circumference, and estimated fetal weight in the INMA-Sabadell cohort
Table S1. Variables included in each fetal growth model in the INMA-Sabadell cohort
Table S2. Characteristics of the study population and average BPA and phthalate metabolites levels (µg/g creatinine) by categories of the different characteristics
Table S3. Correlation matrix of the average BPA and phthalate metabolites levels
Table S4. Sensitivity analyses I: adjusted associations between maternal urinary BPA, MBzP, MnBP, and MiBP levels (µg/g creatinine) and fetal size and growth and birth outcomes adjusting for creatinine and excluding extreme creatinine values
Table S5. Sensitivity analysis II: adjusted associations between maternal urinary BPA, MBzP, MnBP, and MiBP levels (µg/g creatinine) and fetal size and growth and birth outcomes in single- and multi-pollutant models

Table S6. Adjusted associations between maternal urinary ∑DEHPm and its single metabolites (MEHP, MEHHP, MEOHP, MECPP) levels (µg/g creatinine) and fetal size and growth in the overall population

Table S7. Adjusted associations between maternal urinary DEHPm and LMWPm levels (µg/g creatinine) and birth outcomes in the overall population, in girls, and in boys

Table S8. Adjusted associations between maternal urinary MBzP, ∑LMWPm and its single metabolites (MEP, MiBP, MnBP) levels (µg/g creatinine) and fetal size and growth in the overall population

Figure S1. Flowchart of the study population

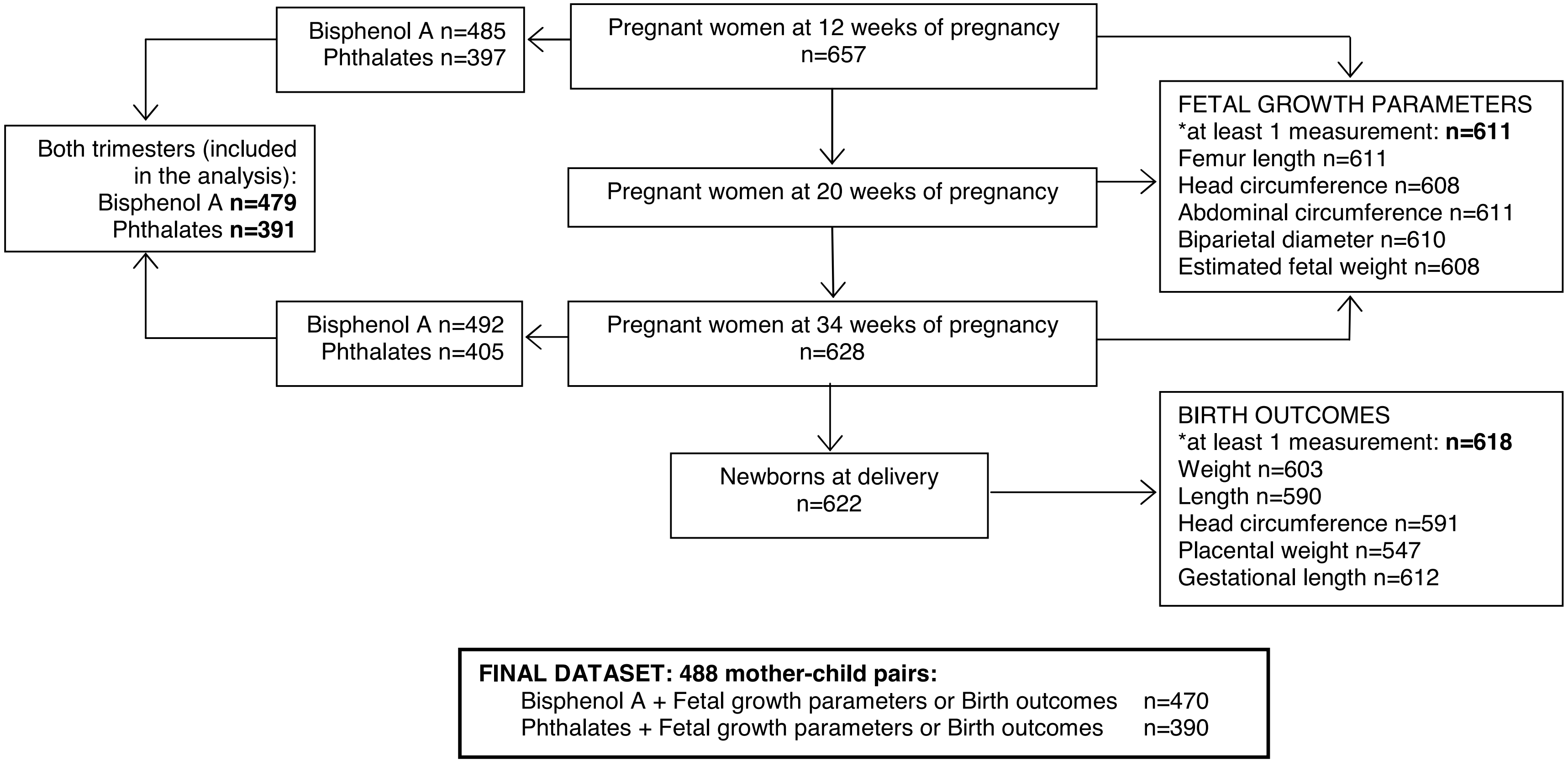

Figure S2. Fetal growth curves for femur length, head circumference, abdominal circumference, and estimated fetal weight in the INMA-Sabadell cohort.

*Each curve represents the growth of a specific ultrasound parameter for a standard child. To construct these curves, continuous variables were included as continuous. In case maternal origin was included in the model, Spanish origin was chosen; in case of parity, nulliparous was chosen. Curves show the growth for a standard boy and a standard girl in case sex was included in the model (see Supplementary Material, Table S1).

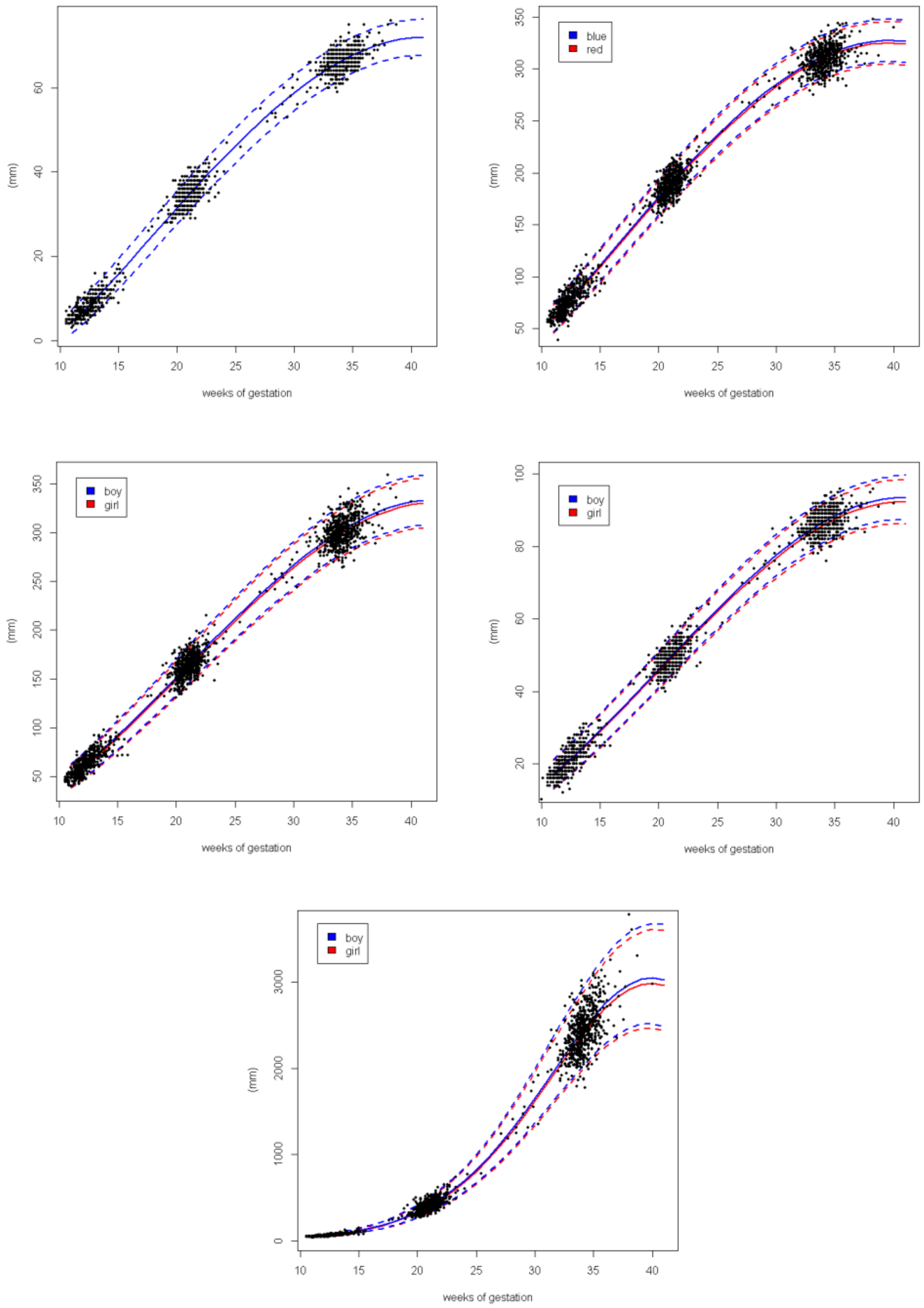

Table S1. Variables included in each fetal growth model in the INMA-Sabadell cohort

	Femur length	Biparietal diameter	Head circumference	Abdominal circumference	Estimated fetal weight
λ	0.80	0.78	0.76	0.59	0.06
P(T) order	3	3	3	3	3
Maternal age	x		x	x	x
Maternal height	x	x	x	x	x
Paternal height					
Maternal weight/BMI			x	x	
Paternal weight/BMI	x	x	x	x	x
Parity			x		
Maternal country of origen	x	x		x	x
Child sex		x	x	x	x
Variance structure	M^{21}, T		M^{30}	parity	M^{30}

Correlation structure was an exponential variogram in all cases and random effects were never incorporated.

Table S2. Characteristics of the study population and average BPA and phthalate metabolites levels[a] (µg/g creatinine) by categories of the different characteristics

**Characteristic**	N with BPA data (%) **(n=470)**	BPA GM (SD)	N with phthalate data (%) **(n=390)**	MEHP GM (SD)	MEHHP GM (SD)	MEOHP GM (SD)	MECPP GM (SD)	MBzP GM (SD)	MEP GM (SD)	MiBP GM (SD)	MnBP GM (SD)
**Child characteristics**											
**Sex**											
Girls	227 (48.2)	2.6 (2.0)	186 (47.7)	11.2 (1.9)	27.9 (1.9)	21.0 (1.9)	40.2 (1.9)	12.6 (2.2)	403.2 (2.6)	33.6 (1.8)	33.9 (2.2)
Boys	243 (51.8)	2.6 (2.0)	204 (52.3)	11.4 (2.0)	30.1 (2.0)	22.4 (1.9)	42.6 (1.9)	12.5 (2.3)	376.7 (2.6)	32.4 (1.9)	31.7 (2.0)
**Birth season**											
Winter	128 (27.2)	2.6* (2.1)	103 (26.4)	11.9 (2.1)	29.7* (1.9)	22.0* (1.8)	42.6* (1.8)	13.0 (2.1)	432.6 (2.7)	31.8* (1.9)	34.5* (2.1)
Spring	123 (26.1)	2.2 (1.8)	100 (25.6)	10.3 (1.9)	25.0 (1.8)	18.9 (1.8)	36.7 (1.7)	12.0 (2.2)	362.3 (2.7)	29.2 (1.8)	30.2 (2.3)
Summer	117 (24.8)	3.2 (2.1)	105 (26.9)	12.5 (2.0)	32.4 (2.1)	24.6 (2.0)	48.3 (2.0)	13.4 (2.4)	393.8 (2.6)	38.1 (1.8)	36.7 (1.9)
Autumn	103 (21.9)	2.6 (1.9)	82 (21.0)	10.6 (1.9)	29.4 (2.1)	21.3 (1.9)	38.1 (2.0)	11.9 (2.3)	366.0 (2.4)	33.2 (2.0)	29.1 (2.0)
**Maternal characteristics**											
**Age (years)**											
<25	37 (7.9)	3.3* (2.1)	25 (6.4)	11.7 (2.0)	30.3 (1.7)	21.8 (1.6)	39.3 (1.6)	12.3 (2.4)	366.9 (2.6)	43.5 (2.0)	36.5 (2.2)
25-29	153 (32.5)	2.7 (2.0)	125 (32.1)	11.3 (1.9)	27.2 (1.9)	20.7 (1.8)	40.2 (1.8)	14.1 (2.2)	395.1 (2.9)	32.5 (1.9)	32.7 (2.0)
30-34	197 (41.8)	2.6 (2.0)	171 (43.9)	11.6 (2.1)	30.1 (2.1)	22.3 (2.0)	42.4 (2.0)	12.1 (2.3)	402.7 (2.5)	32.7 (1.8)	32.4 (2.1)
≥35	83 (17.6)	2.2 (2.0)	68 (17.4)	10.7 (1.8)	28.9 (2.0)	21.6 (2.0)	41.5 (2.0)	11.6 (2.2)	357.6 (2.5)	31.7 (1.9)	32.2 (2.3)
Missings	1 (0.2)		1 (0.3)								
**Pre-pregnancy BMI**											
Underweight	28 (5.9)	2.0 (1.8)	22 (5.6)	10.1 (2.0)	28.4 (1.9)	21.1 (1.8)	37.0 (1.8)	11.7 (2.6)	397.1 (2.6)	33.6 (2.1)	26.2 (2.6)
Normal	314 (66.7)	2.7 (2.0)	267 (68.5)	11.6 (2.0)	28.2 (2.0)	21.0 (1.9)	40.1 (1.9)	11.9 (2.2)	367.5 (2.6)	32.4 (1.9)	32.5 (2.1)
Overweight	90 (19.1)	2.6 (2.0)	70 (18.0)	11.6 (1.8)	31.6 (2.0)	23.6 (1.8)	45.2 (1.8)	15.2 (2.2)	439.8 (3.0)	36.9 (1.9)	37.6 (2.1)
Obese	39 (8.3)	2.5 (1.9)	31 (8.0)	9.7 (2.2)	31.3 (1.9)	23.6 (1.8)	48.7 (2.0)	13.6 (2.2)	475.9 (2.4)	29.7 (1.7)	29.8 (1.8)
**Education**											
Primary	128 (27.2)	2.9 (2.0)	87 (22.3)	13.2* (1.8)	31.7 (1.7)	23.4 (1.6)	45.6 (1.7)	13.4 (1.9)	471.8* (2.7)	34.8 (1.9)	32.1 (1.9)
Secondary	192 (40.8)	2.4 (1.9)	166 (42.6)	10.5	27.3	20.5	39.8	11.6	387.0	30.9	32.8

				(2.0)	(2.0)	(1.9)	(1.9)	(2.3)	(2.4)	(1.8)	(2.2)
University	148 (31.4)	2.6 (2.1)	135 (34.6)	11.2 (2.0)	29.4 (2.1)	22.0 (2.0)	40.7 (2.0)	13.5 (2.4)	349.5 (2.9)	34.8 (2.0)	33.1 (2.1)
Missings	3 (0.6)		2 (0.5)								
**Smoking during pregnancy**											
Never smoke	237 (50.3)	2.5 (2.0)	197 (50.5)	11.1* (2.0)	29.5 (2.0)	22.3 (1.9)	42.9 (1.9)	13.7 (2.3)	377.2* (2.5)	33.1 (1.9)	33.5 (2.0)
Not during pregnancy	161 (34.2)	2.6 (2.0)	136 (34.9)	10.8 (1.9)	27.4 (2.0)	20.2 (1.9)	39.4 (1.9)	11.3 (2.1)	366.7 (2.8)	31.4 (1.9)	30.3 (2.2)
During pregnancy	70 (14.9)	3.0 (1.8)	54 (13.9)	13.8 (2.0)	32.1 (1.9)	23.5 (1.8)	42.9 (1.9)	12.1 (2.1)	542.6 (2.6)	37.3 (2.0)	37.0 (2.2)
Missings	3 (0.6)		3 (0.8)								
**Parity**											
Nulliparous	251 (53.4)	2.8* (2.1)	218 (55.9)	10.6* (2.0)	27.6 (2.0)	20.6 (1.9)	38.6* (1.9)	12.7 (2.3)	362.5 (2.6)	34.1 (1.9)	32.8 (2.0)
Multiparous	217 (46.2)	2.4 (1.9)	170 (43.6)	12.2 (2.0)	30.7 (2.0)	22.9 (1.9)	45.2 (1.9)	12.5 (2.1)	424.5 (2.6)	31.4 (1.9)	32.0 (2.1)
Missings	2 (0.4)		2 (0.5)								
**Urinary cotinine levels during pregnancy**											
Non-SHS exposed (<18 ng/mL)	308 (65.3)	2.4* (2.0)	269 (69.0)	11.1* (2.0)	28.5 (2.0)	21.5 (2.0)	41.0 (1.9)	12.2* (2.3)	364.9* (2.6)	32.2 (1.8)	32.3 (2.1)
SHS exposed (18-50 ng/mL)	67 (14.3)	2.7 (1.8)	49 (12.6)	10.5 (1.6)	28.2 (1.8)	21.8 (1.8)	43.5 (1.7)	16.2 (2.3)	408.8 (2.6)	33.8 (2.0)	33.6 (2.1)
Active smoker (>50 ng/mL)	92 (19.6)	3.1 (1.9)	69 (17.7)	13.4 (2.0)	32.0 (1.9)	22.7 (1.8)	42.2 (1.9)	11.9 (2.0)	505.6 (2.6)	35.5 (2.0)	33.9 (2.1)
Missings	4 (0.9)		3 (0.8)								
**Consumption of canned fish during pregnancy**											
0-0.9	192 (40.8)	2.4* (2.0)	-	-	-	-	-	-	-	-	-
1-1.9	177 (37.6)	2.5 (1.9)	-	-	-	-	-	-	-	-	-
2-7	97 (20.6)	3.3 (2.0)	-	-	-	-	-	-	-	-	-
Missings	5 (1.1)		-	-	-	-	-	-	-	-	-
**Organic food consumption**											
< 1 time/week	-	-	307 (78.7)	11.5 (2.0)	29.4 (2.0)	22.0 (1.9)	42.7 (1.9)	12.5 (2.2)	400.5 (2.6)	32.8 (1.9)	32.4 (2.0)
≥ 1 time/week	-	-	80 (20.5)	11.8 (1.9)	27.9 (2.0)	20.5 (1.9)	37.7 (1.8)	12.8 (2.2)	365.2 (2.7)	33.9 (2.0)	34.0 (2.3)
Missings	-	-	3 (0.8)								
**Use of household cleaning products**[b]											

< 1 time/week	-	-	96 (24.6)	10.4 (2.0)	27.3 (2.0)	20.0 (1.9)	37.6* (1.8)	11.1* (2.2)	309.8* (2.5)	31.2 (1.8)	28.9* (2.1)
≥ 1 time/week	-	-	291 (74.6)	11.7 (2.0)	29.6 (2.0)	22.3 (1.9)	43.0 (1.9)	13.0 (2.2)	423.4 (2.6)	33.7 (1.9)	33.9 (2.1)
Missings	-	-	3 (0.8)								

Abbreviations: BPA: bisphenol A, BMI: body mass index, GM: geometric mean, MEHP: mono-2-ethyl-hexyl phthalate, MEHHP: mono-2-ethyl-5-hydroxy-hexyl phthalate, MEOHP: mono-2-ethyl-5-oxo-hexyl phthalate, MECPP: mono-2-ethyl-5-carboxy-pentyl phthalate, MBzP: mono-benzyl phthalate, MEP: mono-ethyl phthalate, MiBP: mono-isobutyl phthalate, MnBP: mono-n-butyl phthalate, SD: standard deviation, SHS: second-hand tobacco smoke.

[a]Values include concentrations below the LOD for which a value of half the LOD was assigned. Average of measurements at two time points in the first and third trimesters of pregnancy.

[b]Bleach or oven cleaning spray.

*ANOVA *P*-value≤0.1.

Table S3. Correlation matrix of the average BPA and phthalate metabolites levels

	BPA	∑DEHPm	MEHP	MEHHP	MEOHP	MECPP	MBzP	∑LMWPm	MEP	MiBP	MnBP
BPA	1.00										
∑DEHPm	0.22	1.00									
MEHP	0.16	0.78	1.00								
MEHHP	0.21	0.96	0.70	1.00							
MEOHP	0.23	0.97	0.70	0.95	1.00						
MECPP	0.21	0.94	0.65	0.84	0.88	1.00					
MBzP	0.17	0.32	0.20	0.31	0.32	0.30	1.00				
∑LMWPm	0.14	0.16	0.16	0.13	0.14	0.17	0.15	1.00			
MEP	0.13	0.12	0.14	0.10	0.10	0.13	0.10	0.98	1.00		
MiBP	0.12	0.31	0.22	0.33	0.31	0.29	0.30	0.25	0.13	1.00	
MnBP	0.05	0.28	0.21	0.24	0.30	0.27	0.31	0.28	0.15	0.46	1.00

Abbreviations: BPA: bisphenol A, DEHPm: di-2-ethylhexyl phthalate metabolites, LMWPm: low molecular weight phthalate metabolites, MBzP: mono-benzyl phthalate, MECPP: mono-2-ethyl-5-carboxy-pentyl phthalate, MEHHP: mono-2-ethyl-5-hydroxy-hexyl phthalate, MEHP: mono-2-ethyl-hexyl phthalate, MEOHP: mono-2-ethyl-5-oxo-hexyl phthalate, MEP: mono-ethyl phthalate, MiBP: mono-isobutyl phthalate, MnBP: mono-n-butyl phthalate.

Table S4. Sensitivity analyses I: adjusted associations[a] between maternal urinary BPA, MBzP, MnBP, and MiBP levels (µg/g creatinine) and fetal size and growth and birth outcomes adjusting for creatinine and excluding extreme creatinine values

Fetal size and growth	Period		N	Concentrations standardized by creatinine % change (95% CI)	Adjusting for creatinine concentrations % change (95% CI)	N	Excluding extreme creatinine values % change (95% CI)
**BPA**							
FL	weeks 12-20	Overall	452	-4.26 (-7.91. -0.57)	-3.65 (-7.30; 0.04)	415	-3.85 (-7.84, 0.18)
		Girls	220	-1.38 (-7.02, 4.29)	-1.49 (-7.00, 4.04)	199	-1.60 (-7.69, 4.52)
		Boys	232	-5.99 (-10.88, -1.02)	-5.54 (-10.50, -0.48)	216	-5.09 (-10.48, 0.39)
AC	week 12	Overall	452	1.47 (-1.99, 4.92)	1.82 (-1.63, 5.25)	415	1.69 (-2.07, 5.44)
		Girls	220	6.41 (1.16, 11.54)	6.67 (1.55, 11.69)	199	5.41 (-0.46, 11.15)
		Boys	232	-0.75 (-5.35, 3.87)	-0.88 (-5.54, 3.80)	216	0.17 (-4.78, 5.11)
EFW	week 12	Overall	452	1.93 (-1.44, 5.29)	2.21 (-1.15, 5.55)	415	2.55 (-1.12, 6.20)
		Girls	220	5.80 (0.38, 11.11)	5.15 (-0.18, 10.39)	199	5.49 (-0.52, 11.37)
		Boys	232	0.02 (-4.23, 4.28)	0.42 (-3.90, 4.73)	216	1.14 (-3.48, 5.74)
EFW	weeks 12-20	Overall	452	-3.84 (-7.93, 0.30)	-2.60 (-6.70, 1.54)	415	-2.72 (-7.12, 1.71)
		Girls	220	-0.98 (-7.35, 5.42)	-1.09 (-7.31, 5.15)	199	-0.17 (-6.93, 6.60)
		Boys	232	-5.74 (-11.08, -0.29)	-4.36 (-9.81, 1.18)	216	-4.18 (-9.97, 1.70)
**MBzP**							
FL	weeks 20-34	Overall	374	3.70 (0.75, 6.63)	4.03 (1.06, 6.98)	343	3.99 (0.84, 7.11)
		Girls	180	6.12 (1.61, 10.55)	6.38 (1.83, 10.85)	163	6.96 (2.15, 11.67)
		Boys	194	2.10 (-1.86, 6.04)	2.71 (-1.28, 6.68)	180	2.20 (-2.02, 6.39)
**MnBP**							
HC	weeks 12-20	Overall	374	-4.88 (-8.36, -1.36)	-4.73 (-8.24, -1.19)	343	-5.12 (-8.76, -1.44)
		Girls	178	-4.24 (-9.08, 0.66)	-5.08 (-9.84, -0.26)	161	-4.48 (-9.45, 0.57)
		Boys	196	-5.05 (-10.13, 0.11)	-3.74 (-9.03, 1.62)	182	-4.91 (-10.32, 0.60)
AC	weeks 20-34	Overall	374	1.72 (-1.61, 5.04)	2.03 (-1.32, 5.37)	343	1.84 (-1.62, 5.28)
		Girls	180	0.39 (-4.76, 5.54)	0.65 (-4.45, 5.74)	163	0.67 (-4.67, 5.99)
		Boys	194	4.29 (0.01, 8.53)	4.92 (0.51, 9.27)	180	4.56 (0.01, 9.05)
EFW	weeks 12-20	Overall	374	-4.32 (-8.33, -0.27)	-3.99 (-8.04, 0.09)	343	-4.11 (-8.31, 0.13)
		Girls	180	-3.48 (-8.85, 1.96)	-3.77 (-9.06, 1.59)	163	-3.69 (-9.16, 1.86)
		Boys	194	-3.64 (-9.72, 2.53)	-3.23 (-9.48, 3.09)	180	-1.99 (-8.55, 4.63)
EFW	weeks 20-34	Overall	374	2.27 (-0.85, 5.38)	2.63 (-0.52, 5.76)	343	2.29 (-0.98, 5.56)
		Girls	180	1.22 (-3.36, 5.78)	1.37 (-3.17, 5.88)	163	1.40 (-3.37, 6.16)
		Boys	194	4.27 (-0.18, 8.68)	5.19 (0.61, 9.70)	180	4.32 (-0.44, 9.02)

Birth outcomes			N	β (95% CI)	β (95% CI)	N	β (95% CI)
**MBzP**							
Weight	birth	Overall	371	14.11 (-19.08, 47.29)	18.81 (-14.47, 52.08)	341	18.24 (-16.45, 52.93)
		Girls	179	-27.30 (-79.41, 24.8)	-27.40 (-79.67, 24.86)	162	-8.68 (-64.51, 47.15)
		Boys	192	47.78 (5.78, 89.78)	54.48 (12.42, 96.55)	179	44.79 (2.20, 87.39)
Placental weight	birth	Overall	325	0.75 (-9.79, 11.30)	3.04 (-7.56, 13.64)	297	1.32 (-9.78, 12.41)
		Girls	154	-20.57 (-39.22, -1.92)	-17.62 (-36.34, 1.09)	139	-17.01 (-36.70, 2.67)
		Boys	164	12.53 (0.27, 24.79)	14.20 (1.78, 26.62)	158	10.85 (-2.07, 23.76)
**MiBP**							
Weight	birth	Overall	371	-24.83 (-67.05, 17.39)	-13.09 (-54.61, 28.43)	341	-17.42 (-61.27, 26.43)
		Girls	179	-73.16 (-137.16, -9.17)	-51.24 (-114.23, 11.75)	162	-51.39 (-117.66, 14.88)
		Boys	192	19.31 (-35.48, 74.09)	22.94 (-31.62, 77.50)	179	21.93 (-34.91, 78.77)

[a]% change: mean percent difference in standard deviation scores per doubling of BPA levels, Betas: estimated difference in each outcome associated per doubling of exposure levels (levels were log_2-transformed). Femur length models adjusted for maternal age, maternal height, paternal weight, maternal country of origin, maternal education, smoking during pregnancy, parity, birth season, and urinary cotinine levels during pregnancy; Estimated fetal weight model adjusted for maternal age, maternal height, paternal weight, maternal country of origin, maternal education, smoking during pregnancy, parity, birth season, and urinary cotinine levels during pregnancy; Abdominal circumference model adjusted for maternal age, maternal height/weight, paternal weight, maternal country of origin, maternal education, smoking during pregnancy, parity, birth season, and urinary cotinine levels during pregnancy; Head circumference model adjusted for maternal age, maternal height/weight, paternal weight, maternal education, smoking during pregnancy, parity, birth season, and urinary cotinine levels during pregnancy; Birth outcomes models adjusted for maternal education, smoking during pregnancy, parity, birth season, and urinary cotinine levels during pregnancy.

Table S5. Sensitivity analysis II: adjusted associations[a] between maternal urinary BPA, MBzP, MnBP, and MiBP levels (μg/g creatinine) and fetal size and growth and birth outcomes in single- and multi-pollutant models[b]

Fetal size and growth	Period		N	Single-pollutant model % change (95% CI)	Multi-pollutant model % change (95% CI)
**BPA**					
FL	weeks 12-20	All	356	-3.27 (-7.51, 1.01)	-2.77 (-7.17, 1.66)
		Girls	169	-1.93 (-8.49, 4.68)	-1.73 (-8.50, 5.09)
		Boys	287	-3.77 (-9.63, 2.18)	-3.96 (-10.05, 2.23)
AC	week 12	Overall	356	3.19 (-0.63, 6.98)	3.39 (-0.55, 7.30)
		Girls	169	6.36 (0.63, 11.96)	5.80 (-0.12, 11.60)
		Boys	187	2.84 (-2.44, 8.07)	3.24 (-2.25, 8.67)
EFW	week 12	Overall	356	3.28 (-0.45, 6.98)	3.58 (-0.28, 7.40)
		Girls	169	5.50 (-0.31, 11.2)	5.24 (-0.82, 11.18)
		Boys	187	2.8 (-2.19, 7.75)	3.62 (-1.54, 8.72)
EFW	weeks 12-20	All	356	-2.42 (-7.02, 2.22)	-1.60 (-6.36. 3.18)
		Girls	169	-1.00 (-8.19, 6.23)	-0.20 (-7.67, 7.28)
		Boys	187	-3.57 (-9.71, 2.66)	-2.96 (-9.37, 3.54)
**MBzP**					
FL	weeks 20-34	Overall	356	4.10 (1.01, 7.17)	3.79 (0.49, 7.06)
		Girls	169	6.95 (2.07, 11.73)	6.31 (1.29, 11.22)
		Boys	187	2.38 (-1.67, 6.41)	2.10 (-2.39, 6.56)
**MnBP**					
HC	weeks 12-20	Overall	356	-6.53 (-10.05, -2.95)	-6.68 (-10.46, -2.83)
		Girls	167	-6.33 (-11.33, -1.23)	-6.11 (-11.51, -0.60)
		Boys	189	-6.02 (-11.09, -0.86)	-6.88 (-12.40, -1.21)
AC	weeks 20-34	Overall	356	2.11 (-1.31, 5.53)	2.25 (-1.44, 5.93)
		Girls	169	1.47 (-3.92, 6.84)	1.73 (-4.10, 7.52)
		Boys	187	3.84 (-0.53, 8.15)	3.65 (-1.21, 8.46)
EFW	weeks 12-20	Overall	356	-4.80 (-8.99, -0.56)	-4.34 (-8.85, 0.23)
		Girls	169	-4.09 (-9.79, 1.70)	-3.35 (-9.54, 2.93)
		Boys	187	-3.82 (-10.07, 2.51)	-3.70 (-10.63, 3.34)
EFW	weeks 20-34	Overall	356	2.89 (-0.32, 6.08)	0.07 (-0.02, 0.15)
		Girls	169	2.59 (-2.14, 7.28)	0.06 (-0.07, 0.18)
		Boys	187	3.91 (-0.64, 8.41)	3.42 (-1.64, 8.43)
**Birth outcomes**			N	β (95% CI)	β (95% CI)

**MBzP**					
Weight	birth	Overall	354	14.00 (-20.74, 48.75)	11.99 (-25.15, 49.13)
		Girls	169	-32.61 (-88.86, 23.65)	-39.50 (-97.97, 18.97)
		Boys	185	48.05 (5.84, 91.15)	56.31 (9.04, 103.58)
Placental weight	birth	Overall	309	0.23 (-10.57, 11.04)	-1.04 (-12.71, 10.63)
		Girls	145	-21.07 (-40.88, -2.50)	-20.57 (-40.45, -0.69)
		Boys	164	13.02 (0.68, 25.75)	9.31 (-4.95, 23.56)
**MiBP**					
Weight	birth	Overall	354	-19.9 (-63.16, 23.37)	-34.07 (-80.69, 12.55)
		Girls	169	-66.07 (-133.57, 0.10)	-85.77 (-160.95, -10.58)
		Boys	185	21.82 (-33.50, 77.15)	3.05 (-55.57, 61.68)

[a]% change: mean percent difference in standard deviation scores per doubling of BPA levels, Betas: estimated difference in each outcome associated per doubling of exposure levels (levels were log_2-transformed). Femur length models adjusted for maternal age, maternal height, paternal weight, maternal country of origin, maternal education, smoking during pregnancy, parity, birth season, and urinary cotinine levels during pregnancy; Estimated fetal weight model adjusted for maternal age, maternal height, paternal weight, maternal country of origin, maternal education, smoking during pregnancy, parity, birth season, and urinary cotinine levels during pregnancy; Abdominal circumference model adjusted for maternal age, maternal height/weight, paternal weight, maternal country of origin, maternal education, smoking during pregnancy, parity, birth season, and urinary cotinine levels during pregnancy; Head circumference model adjusted for maternal age, maternal height/weight, paternal weight, maternal education, smoking during pregnancy, parity, birth season, and urinary cotinine levels during pregnancy; Birth outcomes models adjusted for maternal education, smoking during pregnancy, parity, birth season, and urinary cotinine levels during pregnancy.

[b]Single-pollutant model after restricting the data to observations with complete data for all four pollutants (BPA, ∑DEHPm, MBzP, and ∑LMWPm). The multi-pollutant model is adjusted for all four pollutants.

Table S6. Adjusted associations[a] between maternal urinary ∑DEHPm and its single metabolites (MEHP, MEHHP, MEOHP, MECPP) levels (μg/g creatinine) and fetal size and growth in the overall population

Parameter/week	∑DEHPm % change (95% CI)	MEHP % change (95% CI)	MEHHP % change (95% CI)	MEOHP % change (95% CI)	MECPP % change (95% CI)
N	374	374	374	374	374
**FL (mm)**					
week 12	-1.15 (-5.05, 2.75)	0.53 (-3.03, 4.08)	-1.35 (-4.86, 2.16)	-1.21 (-4.94, 2.52)	-1.31 (-5.11, 2.51)
week 20	-2.40 (-6.79, 2.02)	-1.56 (-5.57, 2.47)	-2.43 (-6.38, 1.54)	-2.64 (-6.83, 1.59)	-1.38 (-5.68, 2.94)
week 34	1.88 (-2.09, 5.82)	0.67 (-2.94, 4.27)	1.59 (-1.98, 5.14)	1.39 (-2.40, 5.17)	2.08 (-1.79, 5.93)
weeks 12-20	-2.12 (-6.53, 2.32)	-1.90 (-5.92, 2.14)*	-2.08 (-6.04, 1.91)	-2.36 (-6.57, 1.88)	-0.96 (-5.28, 3.37)
weeks 20-34	2.36 (-1.54, 6.23)	0.97 (-2.58, 4.51)	2.07 (-1.44, 5.56)	1.91 (-1.82, 5.62)	2.37 (-1.43, 6.15)
**HC (mm)**					
week 12	0.92 (-3.05, 4.88)	0.69 (-2.93, 4.30)	0.26 (-3.33, 3.84)	0.45 (-3.36, 4.26)	1.20 (-2.67, 5.06)
week 20	0.08 (-4.12, 4.29)	0.27 (-3.56, 4.09)	-0.63 (-4.42, 3.17)	-1.43 (-5.45, 2.60)	0.95 (-3.15, 5.04)
week 34	1.67 (-2.35, 5.66)	-0.44 (-4.09, 3.21)	1.46 (-2.16, 5.07)	1.34 (-2.51, 5.17)	1.98 (-1.93, 5.87)
weeks 12-20	-0.33 (-4.53, 3.88)	-0.01 (-3.84, 3.81)	-0.81 (-4.6, 2.99)	-1.78 (-5.79, 2.25)	0.50 (-3.60, 4.59)
weeks 20-34	1.69 (-2.28, 5.65)	-0.51 (-4.12, 3.11)	1.64 (-1.95, 5.21)	1.69 (-2.13, 5.48)	1.82 (-2.05, 5.67)
**AC (mm)**					
week 12	2.29 (-1.73, 6.30)	2.65 (-1.01, 6.29)	1.83 (-1.79, 5.44)	1.98 (-1.87, 5.82)	1.79 (-2.15, 5.71)
week 20	0.33 (-3.93, 4.59)	-0.34 (-4.22, 3.54)	0.31 (-3.53, 4.14)	-0.18 (-4.26, 3.9)	0.49 (-3.67, 4.65)
week 34	0.65 (-3.33, 4.62)	0.24 (-3.38, 3.86)	0.75 (-2.83, 4.32)	-0.13 (-3.93, 3.68)	0.74 (-3.15, 4.61)
weeks 12-20	-0.78 (-5.12, 3.57)	-1.72 (-5.66, 2.24)	-0.57 (-4.48, 3.34)	-1.19 (-5.34, 2.97)	-0.35 (-4.59, 3.9)
weeks 20-34	0.57 (-3.46, 4.32)	0.32 (-3.24, 3.89)	0.69 (-2.83, 4.20)	-0.10 (-3.84, 3.65)	0.63 (-3.19, 4.45)
**BPD (mm)**					
week 12	1.77 (-2.46, 5.99)	0.25 (-3.60, 4.11)	1.55 (-2.26, 5.34)	1.51 (-2.55, 5.54)	1.93 (-2.20, 6.04)
week 20	1.64 (-2.46, 5.72)	0.07 (-3.67, 3.80)	1.68 (-2.01, 5.35)	1.25 (-2.67, 5.17)	1.36 (-2.64, 5.36)
week 34	-0.93 (-5.25, 3.40)	-2.06 (-5.98, 1.88)	-0.56 (-4.44, 3.34)	-0.19 (-4.33, 3.95)	-1.16 (-5.38, 3.06)
weeks 12-20	0.89 (-3.32, 5.09)	-0.06 (-3.89, 3.77)	1.06 (-2.72, 4.84)	0.60 (-3.43, 4.62)	0.49 (-3.62, 4.59)
weeks 20-34	-1.46 (-5.68, 2.78)	-2.17 (-6.01, 1.68)	-1.08 (-4.88, 2.73)	-0.57 (-4.62, 3.48)	-1.62 (-5.74, 2.52)
**EFW (mm)**					
week 12	0.73 (-3.17, 4.62)	1.98 (-1.57, 5.51)	0.32 (-3.19, 3.83)	0.55 (-3.19, 4.27)	0.33 (-3.48, 4.13)
week 20	-1.21 (-6.11, 3.71)	-1.68 (-6.14, 2.80)	-1.15 (-5.56, 3.28)	-1.56 (-6.24, 3.15)	-0.55 (-5.34, 4.25)
week 34	0.75 (-3.03, 4.52)	-0.41 (-3.85, 3.03)	0.82 (-2.58, 4.21)	0.24 (-3.37, 3.85)	0.91 (-2.78, 4.60)
weeks 12-20	-1.83 (-6.60, 2.96)	-3.11 (-7.43, 1.24)	-1.53 (-5.82, 2.78)	-2.13 (-6.68, 2.45)	-0.83 (-5.50, 3.85)
weeks 20-34	1.17 (-2.51, 4.84)	0.11 (-3.24, 3.45)	1.22 (-2.09, 4.52)	0.75 (-2.77, 4.26)	1.13 (-2.46, 4.71)

Abbreviations: AC: abdominal circumference, BPD: biparietal diameter, CI: confidence interval, DEHPm: di-2-ethylhexyl phthalate metabolites, EFW: estimated fetal weight, FL: femur length, HC: head circumference, MECPP: mono-2-ethyl-5-carboxy-pentyl phthalate, MEHHP: mono-2-ethyl-5-hydroxy-hexyl phthalate, MEHP: mono-2-ethyl-hexyl phthalate, MEOHP: mono-2-ethyl-5-oxo-hexyl phthalate, SD: standard deviation.

[a]Mean percent difference in standard deviation scores per doubling of BPA concentration (levels were log_2-transformed). FL model adjusted for maternal age, maternal height, paternal weight, maternal country of origin, maternal education, smoking during pregnancy, parity, birth season, and urinary cotinine levels during pregnancy; HC model adjusted for maternal age, maternal height/weight, paternal weight, child sex, maternal education, smoking during pregnancy, parity, birth season, and urinary cotinine levels during pregnancy; AC model adjusted for maternal age, maternal height/weight, paternal weight, maternal country of origin, child sex, maternal education, smoking during pregnancy, parity, birth season, and urinary cotinine levels during pregnancy; BPD model adjusted for maternal height, paternal weight, maternal country of origin, child sex, maternal education, smoking during pregnancy, parity, birth season, and urinary cotinine levels during pregnancy; EFW model adjusted for maternal age, maternal height, paternal weight, maternal country of origin, child sex, maternal education, smoking during pregnancy, parity, birth season, and urinary cotinine levels during pregnancy.
*p interaction for sex≤0.1

Table S7. Adjusted associations[a] between maternal urinary DEHPm and LMWPm levels (µg/g creatinine) and birth outcomes in the overall population, in girls, and in boys

	N	Overall – β (95% CI)	N	Girls – β (95% CI)	N	Boys – β (95% CI)	p interaction
**DEHPm**							
**MEHP**							
Weight (g)	371	13.06 (-26.68, 52.80)	179	1.23 (-60.73, 63.18)	192	20.65 (-30.06, 71.36)	0.54
Placental weight (g)	325	2.32 (-9.89, 14.53)	154	-1.39 (-21.95, 19.18)	171	5.19 (-9.69, 20.07)	0.38
Length (mm)	364	1.75 (-0.01, 3.52)	172	1.27 (-1.37, 3.91)	192	2.03 (-0.24, 4.30)	0.37
Head circumference (mm)	359	-0.53 (-1.69, 0.64)	169	-0.96 (-2.77, 0.86)	190	-0.18 (-1.59, 1.22)	0.28
Gestational age (weeks)	375	0.72 (-0.72, 2.17)	180	0.12 (-0.11, 0.34)	195	0.02 (-0.18, 0.21)	0.46
**MEHHP**							
Weight (g)	371	6.30 (-33.53, 46.12)	179	1.41 (-59.59, 62.41)	192	3.04 (-48.83, 54.90)	1.00
Placental weight (g)	325	1.18 (1.18, 13.47)	154	-14.75 (-35.45, 5.96)	171	13.37 (-1.52, 28.25)	0.03
Length (mm)	364	-0.35 (-2.12, 1.42)	172	0.02 (-2.57, 2.62)	192	-0.90 (-3.23, 1.44)	0.76
Head circumference (mm)	359	0.23 (-0.94, 1.40)	169	0.27 (-1.54, 2.07)	190	0.12 (-1.31, 1.55)	0.83
Gestational age (weeks)	375	-0.06 (-1.49, 1.37)	180	0.01 (-0.21, 0.24)	195	-0.03 (-0.22, 0.16)	0.77
**MEOHP**							
Weight (g)	371	10.09 (-32.30, 52.47)	179	-4.17 (-68.23, 59.89)	192	13.26 (-42.61, 69.14)	0.73
Placental weight (g)	325	-0.46 (-13.57, 12.65)	154	-16.11 (-37.65, 5.42)	171	12.80 (-3.43, 29.03)	0.03
Length (mm)	364	-0.19 (-2.08, 1.69)	172	-0.13 (-2.86, 2.61)	192	-0.49 (-3.01, 2.03)	1.00
Head circumference (mm)	359	0.04 (-1.21, 1.29)	169	-0.14 (-2.04, 1.77)	190	0.07 (-1.48, 1.62)	0.63
Gestational age (weeks)	375	-0.14 (-1.67, 1.38)	180	0.02 (-0.21, 0.26)	195	-0.05 (-0.26, 0.15)	0.66
**MECPP**							
Weight (g)	371	19.94 (-23.27, 63.15)	179	9.66 (-53.94, 73.26)	192	21.41 (-37.35, 80.16)	0.75
Placental weight (g)	325	1.98 (-11.29, 15.26)	154	-13.15 (-34.78, 8.48)	171	14.49 (-2.13, 31.11)	0.05
Length (mm)	364	0.55 (-1.37, 2.48)	172	1.32 (-1.40, 4.04)	192	-0.38 (-3.03, 2.28)	0.59
Head circumference (mm)	359	0.18 (-1.11, 1.46)	169	-0.12 (-2.02, 1.78)	190	0.35 (-1.28, 1.98)	0.38
Gestational age (weeks)	375	-0.46 (-2.02, 1.10)	180	-0.01 (-0.24, 0.22)	195	-0.07 (-0.29, 0.14)	0.78
**LMWPm**							
**MEP**							
Weight (g)	371	-5.19 (-32.96, 22.58)	179	-3.26 (-45.97, 39.44)	192	4.62 (-32.28, 41.53)	0.57
Placental weight (g)	325	4.77 (-3.91, 13.44)	154	1.51 (-13.47, 16.49)	171	7.85 (-2.77, 18.47)	0.26
Length (mm)	364	-0.57 (-1.80, 0.67)	172	-0.07 (-1.90, 1.76)	192	-0.69 (-2.35, 0.97)	0.91
Head circumference (mm)	359	-0.32 (-1.14, 0.50)	169	0.08 (-1.18, 1.33)	190	-0.46 (-1.50, 0.58)	0.81
Gestational age (weeks)	375	0.16 (-0.87, 1.18)	180	0.02 (-0.14, 0.18)	195	-0.03 (-0.17, 0.12)	0.46
**MiBP**							
Weight (g)	371	-24.83 (-67.05, 17.39)*	179	-73.16 (-137.16, -9.17)	192	19.31 (-35.48, 74.09)	0.08
Placental weight (g)	325	-1.89 (-14.94, 11.16)	154	-19.54 (-41.07, 1.98)	171	11.21 (-4.81, 27.22)	0.04

Length (mm)	364	-0.83 (-2.71, 1.05)	172	-2.36 (-5.11, 0.39)	192	0.35 (-2.12, 2.83)	0.20
Head circumference (mm)	359	-0.40 (-1.64, 0.85)	169	-1.38 (-3.29, 0.53)	190	0.35 (-1.16, 1.86)	0.19
Gestational age (weeks)	375	0.34 (-1.21, 1.90)	180	0.07 (-0.17, 0.31)	195	-0.02 (-0.23, 0.19)	0.67
**MnBP**							
Weight (g)	371	23.06 (-14.04, 60.15)	179	11.00 (-40.19, 62.20)	192	56.69 (3.40, 109.99)	0.29
Placental weight (g)	325	-4.04 (-15.74, 7.67)	154	-12.80 (-30.92, 5.31)	171	5.97 (-9.33, 21.26)	0.15
Length (mm)	364	1.12 (-0.54, 2.77)	172	0.98 (-1.20, 3.16)	192	2.07 (-0.34, 4.49)	0.91
Head circumference (mm)	359	-0.30 (-1.41, 0.80)	169	-0.69 (-2.24, 0.86)	190	0.69 (-0.80, 2.18)	0.17
Gestational age (weeks)	375	-0.14 (-1.51, 1.22)	180	0.03 (-0.16, 0.22)	195	-0.09 (-0.30, 0.11)	0.35

Abbreviations: CI: confidence interval, DEHPm: di-2-ethylhexyl phthalate metabolites, LMWPm: low molecular weight phthalate metabolites, MECPP: mono-2-ethyl-5-carboxy-pentyl phthalate, MEHHP: mono-2-ethyl-5-hydroxy-hexyl phthalate, MEHP: mono-2-ethyl-hexyl phthalate, MEOHP: mono-2-ethyl-5-oxo-hexyl phthalate, MEP: mono-ethyl phthalate, MiBP: mono-isobutyl phthalate, MnBP: mono-n-butyl phthalate.

[a]Betas represent the estimated difference in each outcome associated per doubling of exposure levels (levels were log_2-transformed). All models were adjusted for maternal education, smoking during pregnancy, parity, birth season, and urinary cotinine levels during pregnancy. Head circumference models also adjusted for type of delivery.

Table S8. Adjusted associations[a] between maternal urinary MBzP, ∑LMWPm and its single metabolites (MEP, MiBP, MnBP) levels (μg/g creatinine) and fetal size and growth in the overall population

Parameter/week	MBzP<br>% change (95% CI)	∑LMWPm<br>% change (95% CI)	MEP<br>% change (95% CI)	MiBP<br>% change (95% CI)	MnBP<br>% change (95% CI)
N	374	374	374	374	374
**FL (mm)**					
week 12	0.19 (-2.79, 3.16)	0.21 (-2.74, 3.15)	0.21 (-2.28, 2.71)	0.20 (-3.60, 4.00)	1.00 (-2.33, 4.32)
week 20	-0.66 (-4.03, 2.71)	-1.28 (-4.61, 2.05)	-0.68 (-3.50, 2.15)	-2.03 (-6.31, 2.27)	-1.92 (-5.67, 1.84)
week 34	3.52 (0.52, 6.49)	-1.29 (-4.27, 1.70)	-1.02 (-3.55, 1.51)	-0.37 (-4.22, 3.49)	2.59 (-0.78, 5.93)
weeks 12-20	-0.78 (-4.16, 2.60)	-1.47 (-4.81, 1.88)	-0.82 (-3.65, 2.02)	-2.28 (-6.57, 2.03)	-2.49 (-6.24, 1.29)
weeks 20-34	3.70 (0.75, 6.63)	-1.08 (-4.02, 1.86)	-0.92 (-3.40, 1.57)	-0.01 (-3.80, 3.79)	2.99 (-0.32, 6.27)
**HC (mm)**					
week 12	0.50 (-2.56, 3.56)	-1.76 (-4.76, 1.26)	-1.25 (-3.79, 1.30)	-0.67 (-4.55, 3.22)	-0.16 (-3.53, 3.2)
week 20	-0.80 (-4.03, 2.45)	-1.49 (-4.68, 1.70)	-0.76 (-3.46, 1.95)	-2.16 (-6.25, 1.96)	-4.50 (-8.00, -0.98)
week 34	0.20 (-2.89, 3.29)*	1.81 (-1.23, 4.85)	1.98 (-0.60, 4.54)	-1.59 (-5.50, 2.34)	-0.11 (-3.50, 3.29)
weeks 12-20	-1.10 (-4.33, 2.14)	-0.85 (-4.03, 2.35)	-0.26 (-2.96, 2.44)	-2.07 (-6.16, 2.04)	-4.88 (-8.36, -1.36)
weeks 20-34	0.38 (-2.68, 3.44)*	2.18 (-0.84, 5.18)	2.18 (-0.36, 4.72)	-1.16 (-5.04, 2.73)	0.89 (-2.48, 4.25)
**AC (mm)**					
week 12	-0.96 (-4.03, 2.11)	-0.79 (-3.83, 2.25)	-0.37 (-2.94, 2.21)	0.47 (-3.45, 4.39)	0.46 (-2.98, 3.89)
week 20	-2.58 (-5.80, 0.66)	-1.77 (-4.97, 1.45)	-0.84 (-3.55, 1.89)	-3.40 (-7.50, 0.74)	-2.82 (-6.42, 0.80)
week 34	-0.48 (-3.51, 2.56)	-0.40 (-3.40, 2.60)	-0.15 (-2.69, 2.38)	-1.60 (-5.46, 2.27)	0.97 (-2.42, 4.35)
weeks 12-20	-2.40 (-5.69, 0.91)	-1.59 (-4.85, 1.69)	-0.76 (-3.53, 2.02)	-4.05 (-8.22, 0.16)	-3.39 (-7.05, 0.30)
weeks 20-34	0.17 (-2.81, 3.15)	0.04 (-2.91, 3.00)	0.06 (-2.44, 2.55)	-0.79 (-4.59, 3.02)	1.72 (-1.61, 5.04)
**BPD (mm)**					
week 12	0.61 (-2.62, 3.84)	-1.32 (-4.51, 1.88)	-0.67 (-3.37, 2.03)	-1.05 (-5.16, 3.07)	-2.22 (-5.80, 1.39)
week 20	-1.42 (-4.54, 1.71)	-2.46 (-5.53, 0.63)	-1.78 (-4.38, 0.84)	-0.89 (-4.87, 3.11)	-2.27 (-5.74, 1.22)
week 34	-1.89 (-5.18, 1.40)	-0.75 (-4.01, 2.51)	-0.28 (-3.05, 2.48)	-1.81 (-6.00, 2.40)	-1.13 (-4.81, 2.55)
weeks 12-20	-1.96 (-5.15, 1.24)	-2.08 (-5.24, 1.09)	-1.66 (-4.33, 1.02)	-0.43 (-4.52, 3.66)	-1.37 (-4.94, 2.22)*
weeks 20-34	-1.56 (-4.78, 1.67)*	-0.06 (-3.26, 3.14)	0.23 (-2.48, 2.93)	-1.63 (-5.74, 2.49)	-0.51 (-4.12, 3.10)
**EFW (g)**					
week 12	-1.05 (-4.01, 1.93)	-0.27 (-3.21, 2.68)	0.02 (-2.47, 2.51)	0.13 (-3.67, 3.92)	0.21 (-3.11, 3.53)
week 20	-2.70 (-6.42, 1.04)	-2.32 (-6.01, 1.39)	-1.22 (-4.35, 1.92)	-3.89 (-8.60, 0.89)	-3.61 (-7.75, 0.56)
week 34	0.38 (-2.50, 3.26)	-1.06 (-3.91, 1.79)	-0.64 (-3.05, 1.77)	-2.27 (-5.92, 1.41)	1.06 (-2.16, 4.27)
weeks 12-20	-2.51 (-6.14, 1.13)	-2.54 (-6.12, 1.07)	-1.43 (-4.48, 1.63)	-4.59 (-9.16, 0.05)	-4.32 (-8.33, -0.27)
weeks 20-34	1.27 (-1.54, 4.06)	-0.37 (-3.15, 2.40)	-0.28 (-2.63, 2.06)	-1.14 (-4.71, 2.44)	2.27 (-0.85, 5.38)

Abbreviations: AC: abdominal circumference, BPD: biparietal diameter, CI: confidence interval, EFW: estimated fetal weight, FL: femur length, HC: head circumference, LMWPm: low molecular weight phthalate metabolites, MEP: mono-ethyl phthalate, MiBP: mono-isobutyl phthalate, MnBP: mono-n-butyl phthalate, SD: standard deviation.

[a]Mean percent difference in standard deviation scores per doubling of BPA concentration (levels were log_2-transformed). FL model adjusted for maternal age, maternal height, paternal weight, maternal country of origin, maternal education, smoking during pregnancy, parity, birth season, and urinary cotinine levels during pregnancy; HC model adjusted for maternal age, maternal height/weight, paternal weight, child sex, maternal education, smoking during pregnancy, parity, birth season, and urinary cotinine levels during pregnancy; AC model adjusted for maternal age, maternal height/weight, paternal weight, maternal country of origin, child sex, maternal education, smoking during pregnancy, parity, birth season, and urinary cotinine levels during pregnancy; BPD model adjusted for maternal height, paternal weight, maternal country of origin, child sex, maternal education, smoking during pregnancy, parity, birth season, and urinary cotinine levels during pregnancy; EFW model adjusted for maternal age, maternal height, paternal weight, maternal country of origin, child sex, maternal education, smoking during pregnancy, parity, birth season, and urinary cotinine levels during pregnancy.
*p interaction for sex≤0.1
